# Fungicidal Behavior of Polyethyleneimine—Polyacrylic Acid Complexes and Hybrids with Silver Nanoparticles: The Role of Filler and Morphology

**DOI:** 10.3390/polym18212772

**Published:** 2026-11-06

**Authors:** Dmitry Igorevich Klimov, Rodion Alexandrovich Vinogradov, Anastasia A. Semenova, Yulia K. Yushina, Anzhelika A. Makhova, Elena V. Zaiko, Maria A. Grudistova, Elena A. Zezina, Alexey A. Zharikov, Alexey A. Zezin

**Affiliations:** 1 Department of Chemistry, M.V. Lomonosov Moscow State University, Leninskie Gory 1-3, Moscow 119991, Russia; vinogr.rodion@gmail.com (R.A.V.); zezi-len@yandex.ru (E.A.Z.); garikov-aleksey@mail.ru (A.A.Z.); 2 Enikolopov Institute of Synthetic Polymeric Materials, Russian Academy of Sciences, Profsoyuznaya st. 70, Moscow 117393, Russia; 3 V.M. Gorbatov Federal Research Center for Food Systems, Talalikhina st., 26, Moscow 109316, Russia; a.semenova@fncps.ru (A.A.S.); yu.yushina@fncps.ru (Y.K.Y.); a.mahova@fncps.ru (A.A.M.); e.zaiko@fncps.ru (E.V.Z.); m.grudistova@fncps.ru (M.A.G.)

**Keywords:** polyelectrolytes, macromolecular complexes, silver nanoparticles, bacterial cells, fungicidal effect, metal–polymer nanohybrids

## Abstract

Interpolyelectrolyte complexes (IPEC) of polyethyleneimine (PEI) and polyacrylic acid (PAA) have inherent biocidal activity, high affinity for various surfaces and can be modified with fillers; therefore, they can serve as platforms for developing aqueous antifungal formulations and prospective surface-treatment systems. IPEC dispersions with PEI/PAA molar ratios of 3/1 and 1/3, as well as PEI/PAA-Ag^+^ complexes, were prepared. UV irradiation of the metal–polymer complexes formed nanoparticles up to 30 nm in PEI-rich and 60 nm in PAA-rich complexes, according to spectroscopy and TEM. Electrophoretic mobility measurements revealed high positive +1.22 ± 0.13 and negative −3.6 ± 0.3 (µm·s^−1^)/(V·cm^−1^) values for 3PEI/1PAA and 1PEI/3PAA IPEC, respectively, which can be attributed to the formation of colloidal particles with an external arrangement of charged loops and tails of the excess component. The antifungal activity of the formulations was evaluated against six mold strains in suspension assays. PEI-rich complexes showed limited activity, whereas PAA-rich IPEC was inactive without silver. Incorporation of silver nanoparticles markedly enhanced activity; 1PEI/3PAA-Ag inhibited all tested mold strains in suspension. In biofilm formation assays, its efficacy reached 99% against *P. polonicum* and 85% against *A. wentii*, respectively. The 3PEI/1PAA-Ag hybrid showed lower efficacy (42% and 87%). Overall, the composites were more effective against developing than preformed biofilms.

## 1. Introduction

Fungal infections and contamination represent a critical challenge to food safety, agriculture and medicine [1,2,3]. Surfaces of equipment and other production facilities are important risk factors because established mold biofilms not only cause food spoilage but may also act as reservoirs for pathogens capable of contributing to large-scale outbreaks of disease. Healthcare facilities are likewise paying increasing attention to the control of microbial contamination because the number of patients susceptible to fungal infections continues to increase. The development of innovative approaches aimed at preventing the dissemination of highly resistant microbial communities, including molds, is therefore of considerable importance. One promising strategy involves the fabrication of abiotic surfaces containing immobilized biocidal agents with prolonged activity. Such materials may be particularly relevant to the food-processing industry and healthcare environments. Silver-containing compounds are among the most important components of antifungal materials [1,2,3,4,5,6,7]. Silver nanoparticles [1] may enter fungal cells and form cytoplasmic aggregates. Their antifungal activity is thought to involve interactions with cellular organelles and proteins, as well as damage to proteins and DNA [1]. The use of silver nanoparticles as antimicrobial agents may also prolong the service life of the formulations because the particles can provide a sustained release of silver ions. In recent decades, the development of polymeric antifungal agents based on silver nanoparticles has received considerable attention due to their high efficacy and broad spectrum of antimicrobial activity, including activity against microorganisms that are resistant to conventional agents [1,2,4,7].

In medicine, considerable efforts are focused on designing wound-healing drugs, modified catheters, materials for dentistry and regenerative medicine with improved fungicidal properties. Candidiasis is a significant health problem that can affect the skin, mucous membranes, and gastrointestinal tract and can cause life-threatening systemic infections [1,3]. Therefore, numerous studies were aimed at combating yeasts of the genus Candida [1,3,8,9,10]. These investigations have mainly focused on the activity of silver nanoparticles immobilized in polymers used in dentistry and ophthalmology [11,12,13,14].

Many other studies were devoted on combating molds to address problems in agriculture and animal husbandry [15,16,17,18,19,20]. In this context, chitosan-based nanocomposites are being extensively studied. For example, composites based on chitosan and silver nanoparticles [15] demonstrated high potential in suppressing *P. expansum*, which often causes infections in dairy cattle. Silver-containing chitosan nanocomposites [19,20] prevented the growth of the mycelia of *Ph. capsici*, *Corynespora cassiicola*, and *F. oxysporum*, leading to increased plant productivity. Another promising field of application for nanoparticles is their immobilization in polymer coatings and antibacterial food-packaging materials [21,22].

It is widely considered that silver nanoparticles are the primary active component in antibacterial metal–polymer nanocomposites. Recent advances in nanoparticle synthesis have demonstrated that the sizes and shapes of nanoparticles may significantly affect their biocidal activity [2,5,23,24]. Numerous works emphasized the high activity of ultrasmall nanoparticles below 5 nm due to their highly values surface area [15,19,24,25]. Meanwhile, there are prominent examples of effective antibacterial and fungicidal materials that include relatively large nanoparticles (30–100 nm, [26,27,28,29,30]). However, the downside of the activity of silver nanoparticles is their potential toxicity. Many works have reported that the use of green methods for nanoparticle synthesis can lead to nanocomposites with reduced toxicity [2,29,30,31,32,33].

Meanwhile, the polymer matrix plays a crucial role regarding the ability to control silver nanoparticle size during synthesis, as well as in terms of antibacterial activity and colloidal stability. When developing biocidal materials, it is important to take into account that macromolecules not only stabilize the nanoparticles but also influence their interactions with cells. Therefore, stabilizers can affect the biocidal activity of nanoparticles. In many cases, macromolecular compounds and metal–polymer nanocomposites provide enhanced antifungal activity combined with reduced toxicity [1,34]. Nowadays, one of the backbone approaches to the preparation of polymeric antibacterial and fungicidal materials is the synthesis of macromolecules containing cationic groups [1,34,35,36]. Cationic compounds bind to the negatively charged cell surface and initiate processes [1,35,37], of cell death, without causing resistance to their effects, unlike antibiotics. Positively charged macromolecules exhibit bactericidal activity against yeast and mold fungi. Increasing attention is being paid to commercially available polymers as their use minimizes the costly development of new formulations.

**Figure 1:**
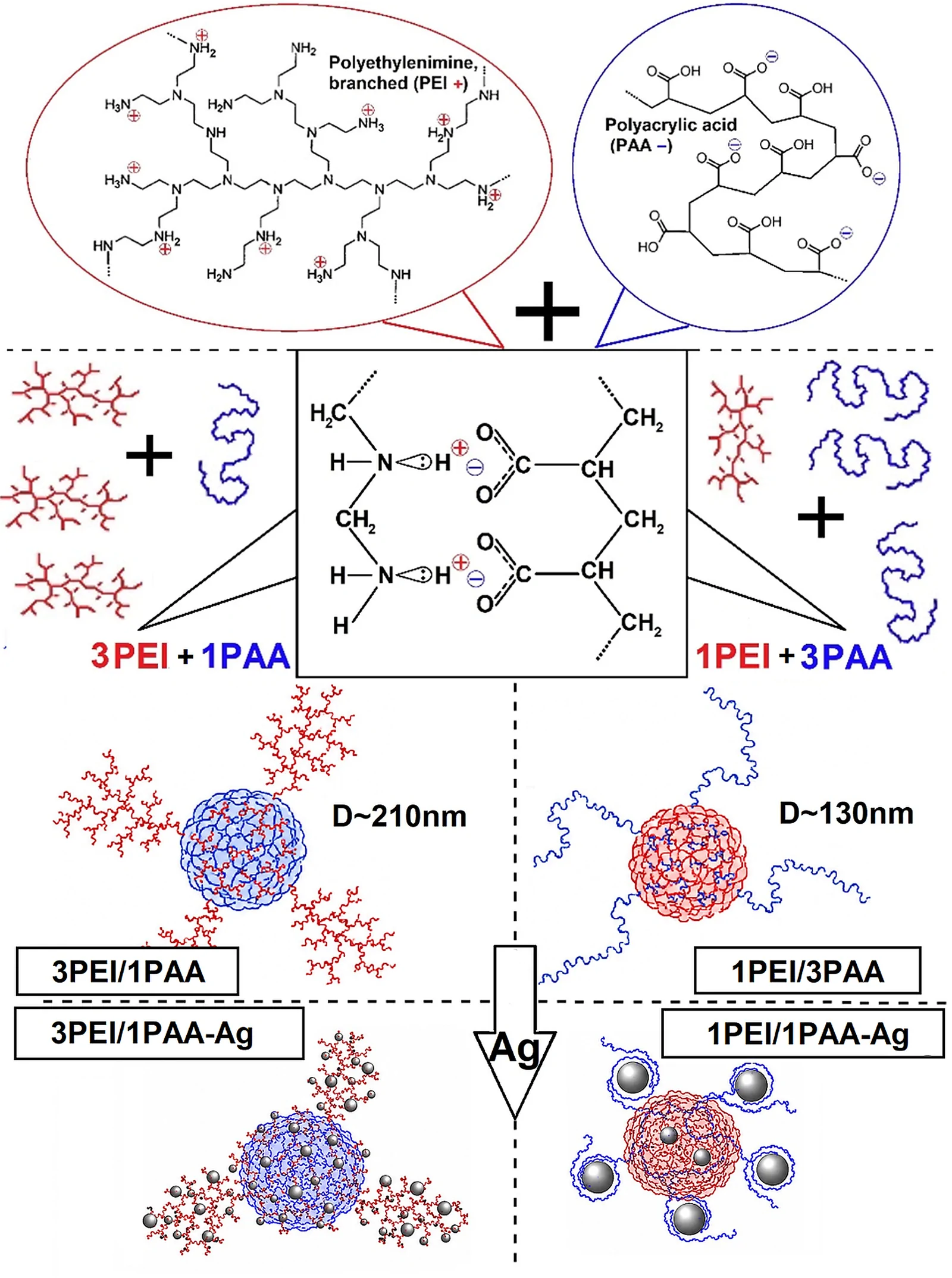
Schematic representation of the formation of non-stoichiometric PEI/PAA interpolyelectrolyte complexes and PEI/PAA-Ag hybrids.

Besides cationic polymers, interpolyelectrolyte complexes (IPECs) formed due to cooperative interactions between oppositely charged units of polyanions and polycations [38] (Figure 1) demonstrate wide potential for use as functional materials, such as flocculants and surface modifiers [38,39]. The ability of polyelectrolytes to bind metal ions opens up prospects for their use as ion-exchange materials or precursors for the synthesis of nanocomposites.

IPECs based on polyethylenimine (PEI) possess intrinsic biocidal activity due to presence cationogenic moieties and have been widely used in the development of antibacterial formulations and abiotic coatings [26,27,40,41,42,43,44]. The use of water as a solvent offers obvious advantages in terms of availability and environmental compatibility. Interpolyelectrolyte complexes can be obtained in soluble or insoluble forms and can be applied as continuous coatings. The relevance of PEI–polyacrylic acid (PAA) complexes is associated with the possibility of tuning their hydrophilic–hydrophobic balance by varying the chemical composition and stoichiometry of the macromolecular complexes. This makes it possible, on the one hand, to adjust the interaction of IPECs with microbial envelopes and thereby impart biocidal properties to the formulations and, on the other hand, to regulate the interaction of polymeric coatings with surfaces of different types [26,27,41,42]. Thus, the complexes can be used to develop abiotic coatings for food storage facilities and medical institutions, they can be bound to soil particles and thus used for plant protection.

The functional groups PAA and PEI are powerful ligands of noble and transition metals [1,34,38,42]. Therefore, PEI/PAA complexes can be combined with metal-containing antimicrobial agents. Films and coatings made of PEI/PAA complexes are known to act as effective stabilizers for nanoparticles [26,27,40,44]. Thus, the antifungal activity of polyelectrolytes may be enhanced by their association with metal ions and nanoparticles. The high complexation power and the ability to stabilize nanoparticles ensure controlled release of silver in various forms. These properties make PEI/PAA IPECs a promising base for multicomponent formulations both in solution and in the form of coating in which high antimicrobial activity is combined with prolonged action. There are numerous examples of the development of antibacterial agents based on PEI/PAA complexes and hybrids with metal nanoparticles [26,27,40,41,42,43].

From the point of view of synthesis, the reduction in metal ions is the general approach for obtaining metal nanostructures in polymer systems. This method allows for the adjustment of nanoparticle sizes by varying the kinetic and thermodynamic parameters of the synthesis, which influence the ratio of nanoparticle nucleation and growth processes. Green methods which include reagent-free approaches (radiation- and photo-initiated reduction in metal ions [19,20,26,27,32,45] and the use in natural substances including polysaccharides [2,29,30,31] for reduction of metal ions are widely used to produce antibacterial and fungicidal materials. The UV irradiation used in this study is an effective and versatile method for producing silver nanoparticles. This method does not require complex equipment and allows for the synthesis of metal–polymer hybrids without impurities [45].

In the present study, non-stoichiometric PEI/PAA complexes with different compositions were prepared, and their morphology was investigated. The formation of silver nanoparticles in UV-irradiated IPEC dispersions was also examined. Commercially available branched PEI and linear PAA were used to prepare the macromolecular complexes. For the first time, a comprehensive comparative assessment of the fungicidal activity of interpolyelectrolyte complexes and metal–polymer hybrids of variable composition based on PEI was carried out. The antifungal properties of the polymer formulations were systematically investigated in mold suspensions, on preformed biofilms and during biofilm formation.

## 2. Materials and Methods

The following reagents were used to prepare the samples: Polyethylenimine (M_w_ = 60,000, practical grade, 30 wt%, aqueous solution) from Serva (Heidelberg, Germany), polyacrylic acid (M_w_ = 100,000, laboratory grade, 35 wt% aqueous solution) from Sigma-Aldrich (St. Louis, MO, USA), and silver nitrate of analytical grade from Reachim (Moscow, Russia). The preparation of dispersions of interpolyelectrolyte complexes was discussed in detail in [26]. In order to obtain metal–polymer complexes, a 0.005 wt% solution of AgNO_3_ was added to a 0.1 wt% dispersion of the PEI/PAA IPEC with a molar ratio of cationic PEI and anionic PAA units 1/3 and 3/1 in low-light conditions with careful stirring, after which the pH values were adjusted to 6 using 0.1 M KOH or H_2_SO_4_ solutions prepared from analytical grade reagents (both manufactured by Reachim, Moscow, Russia).

**Preparation of PEI/PAA IPEC**

Aqueous dispersions of non-stoichiometric PEI/PAA IPEC with PEI/PAA unit ratios of 1/3 and 3/1 were prepared by controlled mixing of dilute polymer solutions at pH 6. All solutions were prepared using deionized water. The pH of the individual polymer solutions was adjusted to 6.0 using 0.1 M KOH or H_2_SO_4_ solutions. The final concentration of the IPEC in all suspensions was 0.1 wt%.

For the preparation of the 1PEI/3PAA IPEC, 50 mL of a 0.2 wt% aqueous PAA solution was mixed with 50 mL of a 0.055 wt% aqueous PEI solution. For the preparation of the 3PEI/1PAA IPEC, 50 mL of a 0.16 wt% aqueous PEI solution was mixed with 50 mL of a 0.10 wt% aqueous PAA solution. The mixtures were stirred for an additional 30 min at room temperature, after which stable IPEC suspensions with a polymer concentration of 0.1 wt% were obtained.

**Preparation of silver-containing complexes**

To prepare the Ag^+^-containing complexes, 20 mL of a 0.13 wt% aqueous AgNO_3_ solution was added to 80 mL of the 0.125 wt% IPEC suspension under stirring. The final Ag-NO_3_ concentration in both cases was 0.005 wt%, corresponding to 50 ppm AgNO_3_ or approximately 31.7 ppm Ag.

The addition of AgNO_3_ was performed slowly under continuous stirring and protection from intense ambient light. In the 1PEI/3PAA–Ag^+^ system, the Ag^+^/PEI functional-group molar ratio was 1/2; in the 3PEI/1PAA–Ag^+^ system, the Ag^+^/PAA functional-group molar ratio was 1/2.5.

**Preparation of silver nanoparticles-containing hybrids**

Silver nanoparticles were formed by photoreduction of Ag^+^ ions in the polymer complexes. The 0.1 wt% suspensions of 1PEI/3PAA–Ag^+^ and 3PEI/1PAA–Ag^+^ were irradiated once for 20 min using an OUFL-01 UV emitter equipped with a DKBU-7 lamp (effective emission range 180–275 nm). During irradiation, 30 mL portions of the suspensions were placed in glass flasks and continuously stirred. The distance between the UV source and the liquid surface was 3 cm. After irradiation, the samples were denoted 1PEI/3PAA–Ag and 3PEI/1PAA–Ag, respectively.

**Instrumental Characterization**

The structure of the nanocomposite material was studied using the transmission electron microscope (TEM) “Leo-912 AB OMEGA” (Carl Zeiss, Oberkochen, Germany) with a resolution of 0.3 nm, equipped with an in-column OMEGA energy filter and a LaB_6_ electron source. The measurements were carried out at an accelerating voltage of 100 kV. Data on the size and spatial distribution of the nanoparticles were obtained from the analysis of about 150–300 objects in the TEM images. UV-Vis spectra and turbidimetric data were measured using a Perkin Elmer Lambda 9 spectrometer with an optical range of 200–900 nm (Überlingen, Germany). Measurements were performed in a quartz cuvette with an optical path length of 1 mm.

FTIR measurements were performed in transmission mode using an INFRA-SPEK FSM 2201 FTIR spectrometer. The spectra were acquired over the range of 400–4000 cm^−1^ at a resolution of 4 cm^−1^, using 64 scans. To investigate the structure of the interpolyelectrolyte and metal–polymer complexes, films were prepared from aqueous polymer dispersions on the surfaces of germanium plates.

Mean hydrodynamic diameters of complexes were obtained by dynamic light-scattering (DLS) using Brookhaven ZetaPlus equipment (Brookhaven, GA, USA) in a thermostatic cell. The analysis of the data was performed with the software supplied by the manufacturer. Additionally, the autocorrelation functions were analyzed using DynaLS program. Electrophoretic mobility (EPM) of complexes was determined by laser microelectrophoresis using Brookhaven ZetaPlus equipment (Brookhaven, GA, USA) in a thermostatic cell. The analysis of the data was performed with the software supplied by the manufacturer.

Potentiometric monitoring of Ag^+^ was performed using an Anion 4100 ion meter (IzTech, Moscow, Russia), an Elis-131Ag silver ion-selective electrode (IzTech, Moscow, Russia), and a Re-10108 reference electrode (IzTech, Moscow, Russia). To determine the concentration of free silver ions, the following equation was used: E = E_0_ + b log_10_ a(Ag^+^) [46], where E is the measured electrode potential, a(Ag^+^) is the activity of Ag+ ions. A preliminary calibration of the constant parameters (E0 and b) for a two-electrode device system was performed.

**Antifungal activity**

The antifungal activity of PEI/PAA-based polymer complexes against molds was evaluated in three stages: using a modified quantitative suspension assay and in models of preformed and developing mold biofilms (more detailed information in Supplementary Materials, Scheme S1). Six mold strains isolated from cheese-processing facilities and identified by specialists of the V. M. Gorbatov Federal Research Center for Food Systems were used in the suspension assay:

• *Penicillium gladioli* No. 7034.
• *Penicillium rugulosum* No. 7040.
• *Penicillium polonicum* No. 7046.
• *Cladosporium pseudochalastosporoides* No. 7036.
• *Botrytis cinerea* No. 7045.
• *Aspergillus wentii* No. 7049.

Two of the six strains, *A. wentii* and *P. polonicum*, were selected for subsequent experiments involving preformed and developing biofilms.

For the suspension assay, 4.5 cm^3^ of each polymer-complex dispersion was prepared in sterile tubes. In parallel, mold suspensions were prepared by cultivating the test strains for 5 days on Sabouraud medium supplemented with chloramphenicol. The microorganism suspensions were then mixed with the polymer formulations and incubated for 24 h. The criterion for the manifestation of fungicidal activity was considered to be 100% death of mold fungi. Complete inhibition was defined as the absence of detectable growth under the applied experimental conditions.

A glass-fiber-filter biofilm model was used to evaluate the activity of the polymer complexes against preformed mold biofilms. The test strains were cultivated on Sabouraud agar (Federal State Budgetary Scientific Institution “State Research Center for Applied Microbiology and Biotechnology” [SRCAMB], Obolensk, Russia) for 5 days at 24 °C until pronounced sporulation was observed visually. Then, 10 cm^3^ of sterile buffered peptone water (BPW, pH 7.0) was applied to the agar surface, and the spores were gently collected with a sterile spatula. To obtain a homogeneous suspension and remove large mycelial fragments, the material was filtered through sterile homogenization bags containing a nonwoven filter.

The resulting suspension was used to form biofilms. Glass-fiber filters were placed on the surface of Sabouraud agar in Petri dishes, and 20 µL of the suspension of the corresponding test strain was applied to each filter. The samples were incubated for 24 h at 24 °C. After biofilm formation, 40 µL of the corresponding polymer-complex dispersion was applied to each filter, followed by an additional 24 h incubation at 24 °C. Sterile distilled water was used instead of the test-strain suspension in the negative control. All experiments were performed in triplicate.

The initial concentration of viable cells in the working suspension was determined by serial tenfold dilution. Dilutions of 10^−1^ and 10^−2^ were plated onto Sabouraud agar in a volume of 1 cm^3^, with three parallel plates prepared for each dilution. The plates were incubated for 5 days at 24 °C, after which the number of mold colonies was counted. The results were expressed as CFU cm^−3^ and calculated as the arithmetic mean of three parallel measurements. The standard deviation in all experimental series ranged from 0.10 to 0.36.

The antifungal efficacy of the polymer complexes on treated surfaces was evaluated from the reduction in viable-cell numbers relative to the untreated control. The number of colonies recovered from the control surfaces was defined as 100%.

The percentage reduction was calculated using the following equation:

$$X=\left(1-\frac{N_{\mathrm{treated}}}{N_{\mathrm{control}}}\right)\times 100\%,$$

where *N*_treated_ is the number of viable microbial cells recovered from the treated surface, expressed as CFU cm^−3^, and *N*_control_ is the number of viable cells recovered from the untreated control surface, expressed as CFU cm^−3^.

An efficacy of 0% and negative values were interpreted as the absence of an inhibitory effect.

The antifungal efficacy of the polymer complexes during biofilm formation was then evaluated. The test-strain suspensions were prepared as described above. In this experimental series, the glass-fiber filters were pretreated with 50 µL of the corresponding polymer-complex dispersion and dried at room temperature for 30 min. The polymer-treated filters were placed aseptically on Sabouraud agar in Petri dishes, and 15 µL of the suspension of the corresponding test strain was applied to each filter. The samples were incubated for 5 days at 24 °C in triplicate. After incubation, viable cells were enumerated by plating, and the antifungal efficacy of the polymer formulations was calculated using the same procedure as that used for the preformed-biofilm assay.

Thus, the antifungal properties of the PEI/PAA complexes were assessed in mold suspensions, against preformed biofilms and during biofilm formation. This approach made it possible to characterize both the direct antifungal action of the polymer formulations and their ability to prevent mold development and survival in the biofilm state.

**Statistical analysis**. All experiments were performed in triplicate, and the results are presented as mean ± standard deviation. For the biofilm assays, differences in viable-cell counts between treated samples and the corresponding untreated controls were assessed using Student’s *t*-test. Differences were considered statistically significant at *p* < 0.05.

## 3. Results and Discussion

### 3.1. Preparation and Characterization of PEI/PAA Complexes and Ternary PEI/PAA-Ag Nanoparticle Hybrids

To study the fungicidal properties, IPEC and metal–polymer hybrids were prepared, both with an excess of PEI and with an excess of PAA. Non-stoichiometric macromolecular complexes of branched PEI and linear PAA are water-dispersible at the colloidal level. Their formation and morphology depend on several factors, including pH, ionic strength, molecular weight, concentration and the ratio of the polyanionic and polycationic components. The colloidal particles may incorporate several macromolecules and possess a complex internal organization. Cooperative interactions between negatively and positively charged groups leads to the mutual neutralization of charges and the formation of hydrophobic extended regions (Figure 1), whereas excess polymer units form charged loops and tails [38,47].

**Figure 2:**
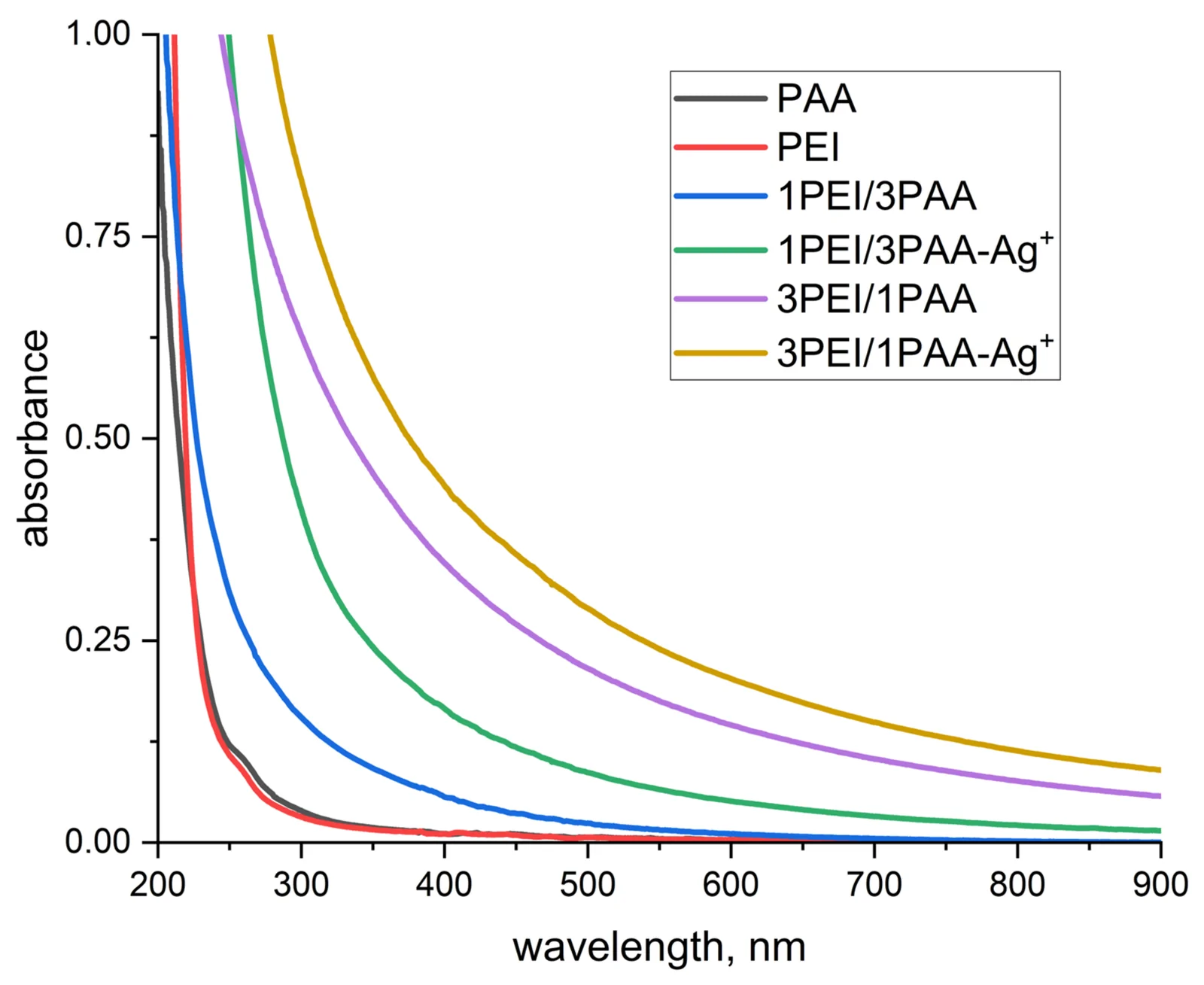
UV-Vis spectra of PAA and PEI solutions, interpolyelectrolyte and metal–polymer complex dispersions.

For the development of effective biocidal formulations, it is important to consider not only the content of cationic groups but also the morphology of the colloidal particles. For comparative experiments, IPEC dispersions containing either an excess of PEI units or an excess of PAA units were prepared (3PEI/1PAA and 1PEI/3PAA). PEI and PAA were soluble in water and formed transparent solutions (Figure 2).

The addition of PEI solution to PAA solution or PAA solution to PEI solution resulted in turbidity. This behavior is typical for mixtures of oppositely charged polyelectrolytes [38] and indicates the formation of IPEC particles. Such complexes were not molecularly soluble in water but remained dispersed at the colloidal level because of an excess of positively or negatively charged ionogenic groups. For obtaining IPEC, a pH value of 6 is optimal, as in this case the degree of protonation of PEI is relatively high (0.41 [7]), and the degree of dissociation of the PAA units is 0.44 [48]. An increase or decrease in acidity leads to lowering in one or the other value.

As in previous work [26] IPECs with PEI/PAA unit ratios of 3/1 and 1/3 were selected for the preparation of ternary polyanion–metal ion–polycation complexes because these compositions showed relatively high aggregative stability. The PEI/PAA IPECs were characterized by dynamic light scattering and laser microelectrophoresis. The IPEC with an excess of the polycation had an average diameter of 210 nm, a PDI of 0.192 and an electrophoretic mobility of +1.56 ± 0.11 (µm·s^−1^)/(V·cm^−1^). The IPEC with an excess of the polyanion had an average diameter of 130 nm, a PDI of 0.171 and an electrophoretic mobility of −2.97 ± 0.14 (µm·s^−1^)/(V·cm^−1^) (more detailed information in Supplementary Materials, Table S1).

These results show that the sign of electrophoretic mobility is determined by the component present in excess. Thus, we can conclude that the hydrophobic regions (the PAA and PEI charges neutralize each other) are localized in the more compact internal parts of the complexes, while the uncompensated units of the excess component form external loops and tails that contribute to colloidal stability (Figure 1).

Previous studies showed that PEI/PAA IPEC coatings and dispersions containing silver nanoparticles exhibited substantially higher biocidal activity against Gram-negative and Gram-positive bacteria than the corresponding PEI/PAA macromolecular complexes [26,27]. To investigate whether the antifungal activity of the IPECs could be enhanced, hybrids containing silver nanoparticles were prepared. In the first stage, metal–polymer complexes (PEI/PAA-Ag^+^) have been synthesized as precursors for nanohybrids (PEI/PAA-Ag).

The metal–polymer complexes were prepared at pH 6 because, under acidic conditions, the efficiency of silver-ion complexation with the functional groups of PAA and PEI may decrease due to competition between metal-ion binding and protonation of the IPEC functional groups [26]. Adding a silver nitrate solution to IPEC dispersions leads to an increase in their turbidity (Figure 2) due to the formation of intermolecular bonds, which qualitatively demonstrates the formation of metal–polymer complexes. However, the turbidity of the dispersions does not allow us to draw conclusions about the nature of metal ion binding.

**Figure 3:**
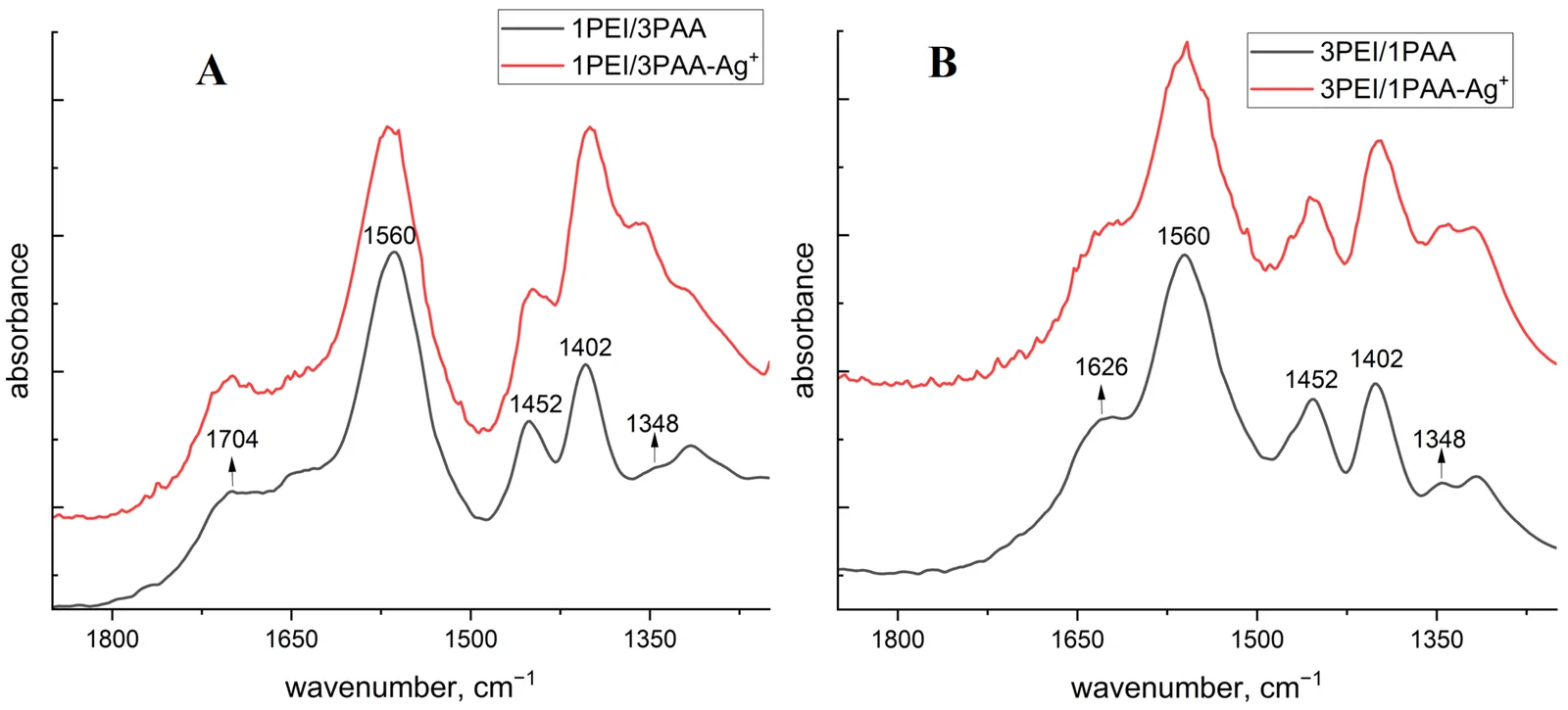
FTIR spectra of PEI/PAA interpolyelectrolyte complexes and PEI/PAA-Ag^+^ complexes with PEI/PAA unit ratios of (**A**) 3/1 and (**B**) 1/3.

FTIR spectroscopy (Supplementary Materials, Figure S1) was used to obtain information about metal–polymer complex formation. The spectral region from 1800 to 1350 cm^−1^ (Figure 3) contains signals of ionized and non-ionized carboxylate and nitrogen-containing groups [49,50,51,52,53] of macromolecular complexes and therefore was particularly informative for analyzing complex formation. When analyzing experimental data, it should be taken into account that the positions of the band maxima and their widths in the spectra of PAA, PEI, and IPECs [49,51,52,54] depend significantly on the method of the sample preparation due to the effect of intermolecular interactions. The spectra contained bands assigned to asymmetric and symmetric stretching vibrations of the carboxylate anion with a maxima at approximately 1560 and 1402 cm^−1^, and of non-ionized carboxylic groups (1704 cm^−1^) [49,51,52]. Bands near 1348 and 1626 cm^−1^ were assigned to vibrations of N–H-containing groups [50,53]. It should be noted that the broad PAA band with a maximum at 1560 cm^−1^ may also contain a small signal corresponding to the deformation vibrations of the -NH group in PEI, and the intensity and position of the PAA band with a maximum at 1402 may be slightly influenced by the valence vibration of the C-N bond [49,50,52].

The band at 1452 cm^−1^, attributed to deformation vibrations of the –CH_2_—groups [49,54], was used for comparison of the FTIR spectra of macromolecular complexes with different compositions. In samples with PEI/PAA unit ratios of 3/1 and 1/3, the incorporation of silver ions resulted in a substantial increase in the intensity of the signals at approximately 1348 both 1560 and 1402 cm^−1^, assigned to nitrogen-containing and carboxylate groups, respectively (Figure 3), supporting the formation of triple PEI/PAA-Ag^+^ complexes. The conclusion about the formation of a metal–polymer complex is confirmed by the fact that the complexation of PEI with copper ions led to similar effects [50] for the bands in ranges 1315–1345 and 1580–1650 cm^−1^ and for PAA band at 1560 cm^−1^ of the PEI- PAA complex when nickel ions were included [55].

**Figure 4:**
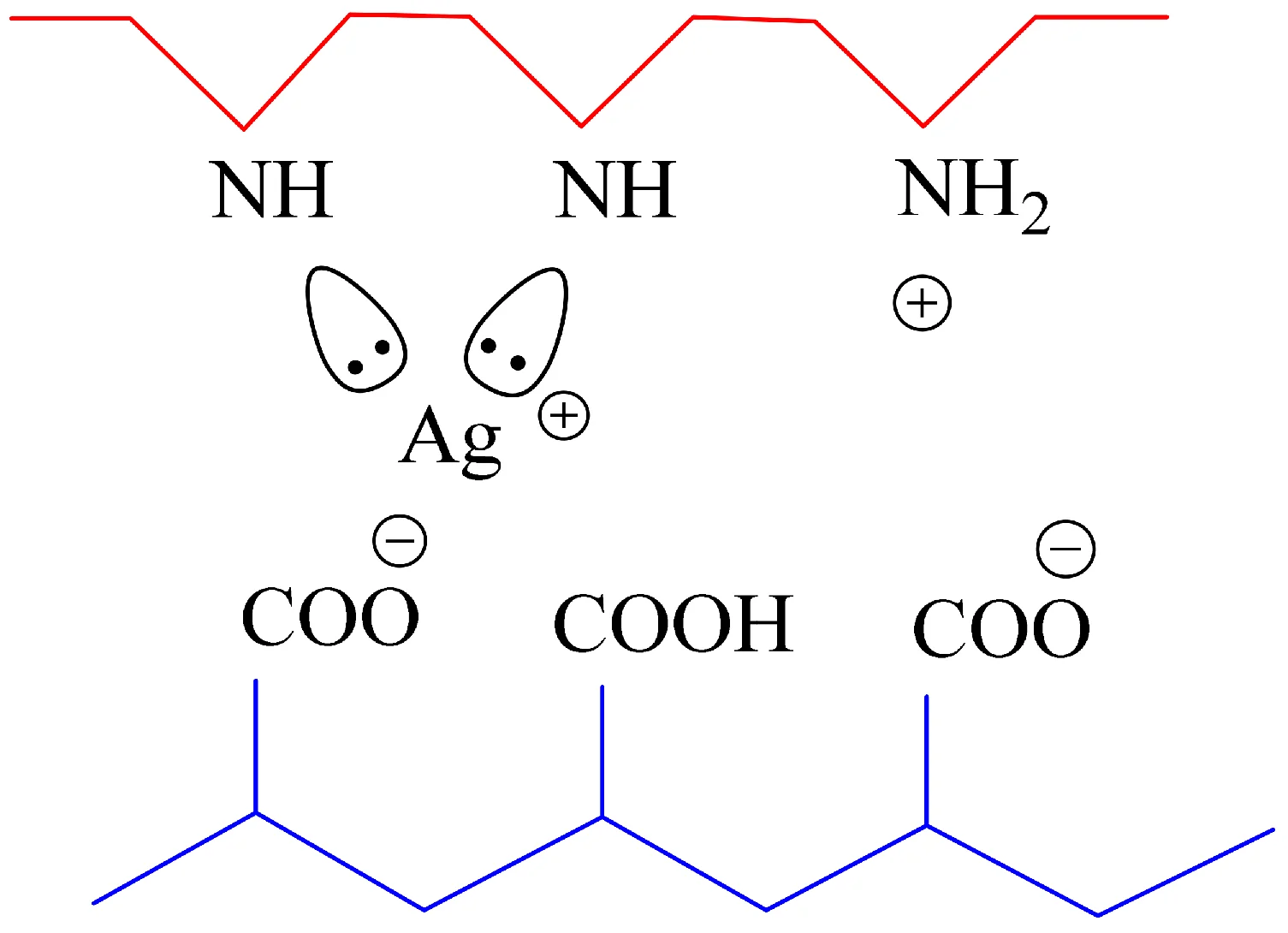
Proposed structure of the PEI/PAA-Ag^+^ metal–polymer complexes.

EPR spectroscopy previously showed [38] that, in stoichiometric PEI/PAA IPEC films, metal ions can be incorporated between the cationic and anionic polymer chains, forming a “sandwich-like” structure that increases the stability of ternary metal–polymer hybrids. In the triple PEI/PAA-Ag^+^ complexes (Figure 4), silver ions are coordinated by the nitrogen-containing groups of PEI and interact ionically with the carboxylate groups of PAA.

**Figure 5:**
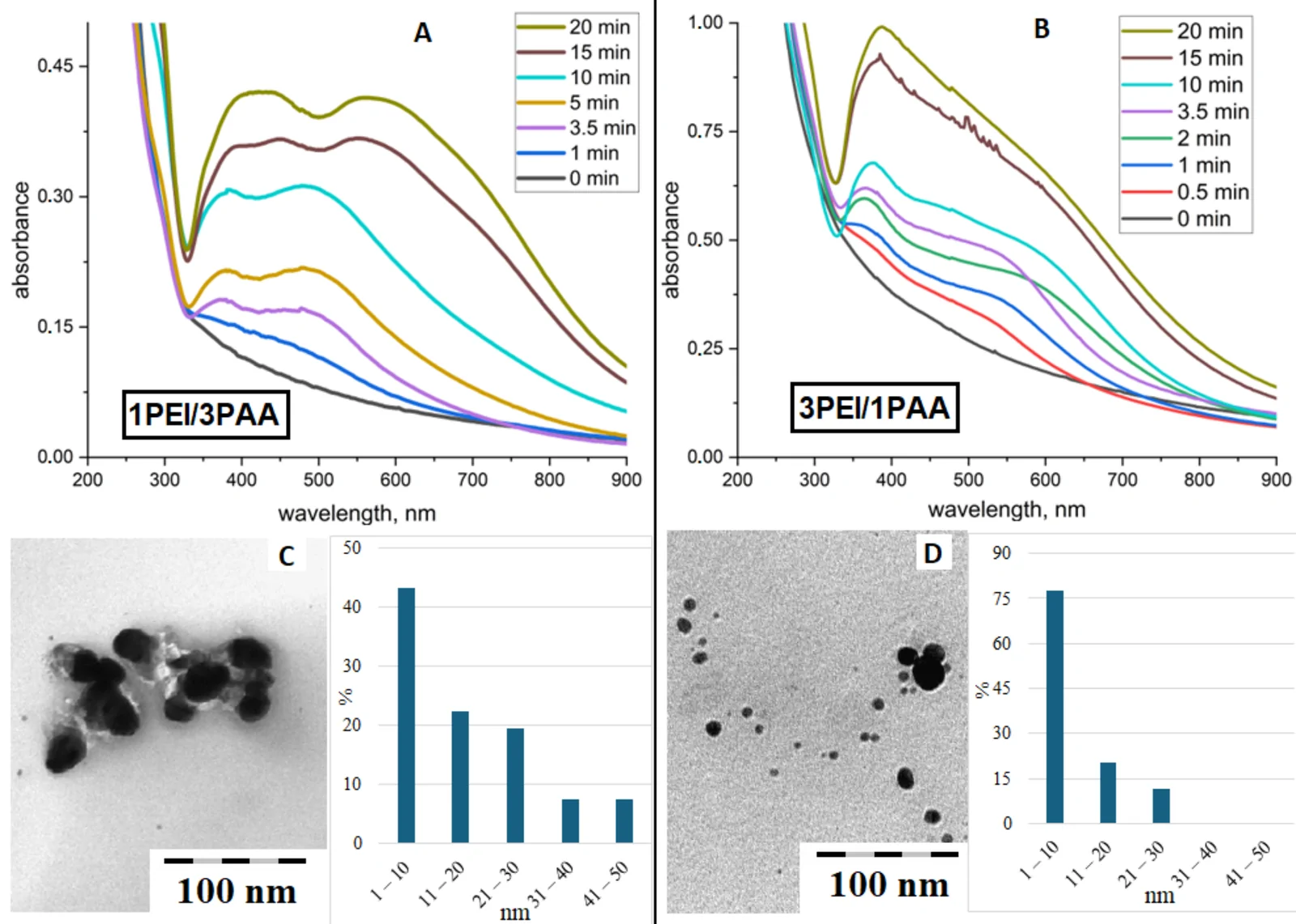
UV–Vis spectra of irradiated PEI/PAA-Ag^+^ complexes with the molar ratio of PEI/PAA units equal to (**A**)—1/3; (**B**)—3/1. Micrography and nanoparticles size distribution in systems with molar ratio of PEI/PAA units equal to (**C**)—1/3; (**D**)—3/1.

UV irradiation of dispersions of metal–polymer complexes was used for the controlled synthesis of nanoparticles. The optical spectra of the PEI/PAA-Ag^+^ complexes after different irradiation times are shown in Figure 5. For example, after 1 min of irradiation, the spectra of the complexes showed two distinct absorption bands: a short-wavelength band with a maximum at approximately 390 nm and a long-wavelength band with a maximum near 550 nm (Figure 5A,B). Both these bands can be attributed to the surface plasmon resonance of silver nanoparticles. As the irradiation time increased, the integrated spectral intensity first increased and then reached a plateau after approximately 15–20 min, indicating that the reduction of silver ions was approaching completion. In addition to the increase in band intensity, the absorption maxima shifted towards longer wavelengths. The maximum of the short-wavelength band shifted from approximately 380 to 420 nm, whereas the maximum of the long-wavelength band shifted from approximately 550 to 600 nm. The long-wavelength band gradually developed into a shoulder. Further irradiation for more than 20 min caused a gradual increase in optical density throughout the 500–900 nm region, indicating light-induced aggregation of the nanoparticles.

For the complex with an excess of PAA, increasing the irradiation time to 15 min resulted in three weakly resolved maxima at approximately 375, 440 and 570 nm. Together with intense scattering in the long-wavelength region from 700 to 900 nm, these features may indicate the formation of larger nanoparticles and nanoparticle aggregates in the 1PEI/3PAA complex (Figure 5A) than in the 3PEI/1PAA complex (Figure 5B).

Indeed, according to Mie theory, the optical spectra of non-aggregated sols of spherical silver nanoparticles with sizes ranging from a few to several tens of nanometers are characterized by a single intense band with a maximum in the ca. 380–440 nm region, corresponding to the excitation of the dipolar mode of surface plasmon resonance [56]. However, for larger nanoparticles, aggregates, and particles with anisotropic shapes, the plasmon resonance spectra may contain more than one band due to several different mechanisms [56]. For relatively large silver nanoparticles (with sizes above approximately 80–100 nm), the appearance of a second band is associated with the excitation of the quadrupolar plasmon resonance mode [57]. In this case, the quadrupolar peak appears near the original position of the dipolar one, while the latter shifts toward longer wavelengths [56]. Further increasing the nanoparticle size can lead to the appearance of additional bands, resulting in optical spectra comprising three or more poorly resolved peaks [57]. For nanoparticles with anisotropic shapes, such as nanorods, splitting of the plasmon absorption band may result from the excitation of longitudinal and transverse dipolar modes [56]. In nanoparticle aggregates, the nature of the spectral splitting and the overall spectral shape depend on various parameters, including the characteristic aggregate size, aggregate shape, and interparticle distance, among others. In the simplest case of two spherical nanoparticles of identical size, plasmon coupling depends directly on the interparticle distance and increases sharply as the particles approach each other. In optical spectra, this effect initially manifests itself as broadening of the single plasmon band. As the interparticle distance decreases, a shoulder appears on the long-wavelength side of the band, eventually leading to the splitting of the peak into two components [58,59].

To correlate the experimentally observed changes in the optical spectra with the sample morphology, the irradiated complexes were examined by TEM (Figure 5C,D and Supplementary Materials, Figures S2 and S3). The irradiated complex with an excess of PEI (3PEI/1PAA) contained predominantly nanoparticles with sizes of 5–20 nm (Figure 5D). For non-aggregated nanoparticles in this size range, the plasmon spectrum typically consists of a single band. However, TEM images reveal not only individual nanoparticles but also clusters of closely spaced nanoparticles, including pairs. Thus, the spectrum shown in Figure 5B appears to result from the combined contributions of non-aggregated nanoparticles and their aggregates. On the contrary, the irradiated complex with an excess of PAA (1PEI/3PAA) contained a fraction of nanoparticles larger than 60 nm, as well as nanoparticle aggregates (Figure 5C). Figure 1 shows a schematic representation of the obtained metal–polymer nanohybrids 3PEI/1PAA-Ag and 1PEI/3PAA-Ag. Thus, the UV-Vis spectroscopy and TEM findings suggest a lower nanoparticle-stabilizing capacity of the PAA-rich complex. At the same time, the lower degree of surface passivation may favor the antifungal activity of the silver nanoparticles.

According to the general understanding of the overall mechanism of photochemical reduction in metal ions [60] under UV exposure, the first stage (reaction 1) involves the generation of excited states of silver ion complexes, in which the ligands are the functional groups of PAA and PEI:

(1) [PEI—Ag^+^—PAA] + hν → [PEI—Ag^+^—PAA]*

Furthermore, metal complexes with ligand-to-metal charge-transfer excited states undergo electron transfer (reaction 2), resulting in the formation of silver atoms:

(2) [PEI—Ag^+^—PAA]* → [PEI—Ag^0^—PAA]^+^

The subsequent reactions involve the formation of a number of quasi-molecular clusters that act as seeds for nanoparticles (reaction 3).

(3) Ag_0_ + Ag^+^ → Ag_2_^+^ → … → (Ag_m_)^n+^

The stabilizing matrix has a significant effect on the mechanism of nanoparticle formation. Thus, during the photochemical reduction of silver ions in the presence of macromolecules of partially dissociated polyacrylic acid, a stable bright blue coloration was observed at the early stages, which was attributed to the stabilization of silver clusters characterized by a broad absorption band with a maximum at 700–750 nm [61]. This coloration of the solutions is specific to silver clusters stabilized by macromolecules of partially dissociated polyacrylic acid and has also been observed during radiation-chemical and borohydride reduction of Ag^+^ ions.

However, it should be emphasized that the absence of such coloration at the initial stages of photoreduction of silver ions, even in 1PEI/3PAA complexes, indicates specific conditions for the formation of nanoparticles in the triple PEI/PAA-Ag^+^ complex. At the same time, the formation of much smaller silver nanoparticles during the photoreduction of 3PEI/1PAA complexes indicates a significantly higher efficiency of the nucleation stage compared to 1PEI/3PAA complexes at the same initial concentration of Ag^+^. Furthermore, as clusters and nanoparticles form, polyethyleneimine may participate in the chemical reduction of silver ions adsorbed on the metal surface.

**Figure 6:**
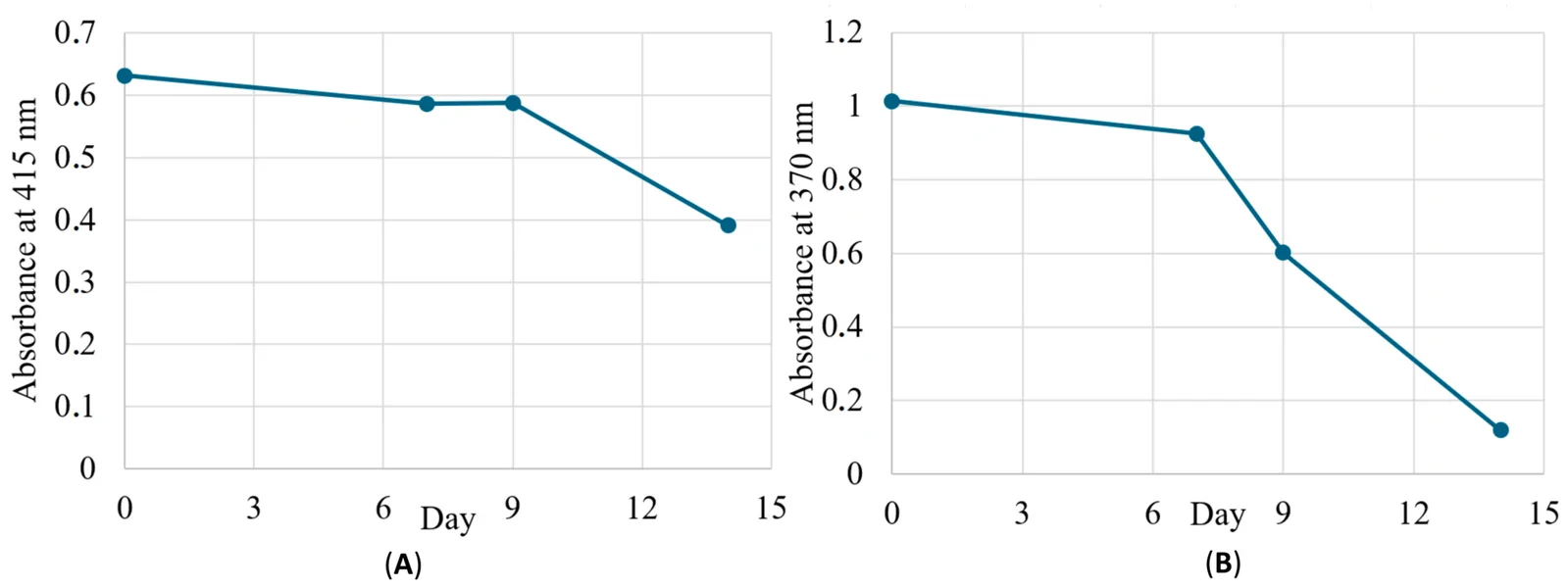
Monitoring of colloidal stability of 1PEI/3PAA-Ag (**A**) and 3PEI/1PAA-Ag (**B**) dispersions according to UV-Vis spectroscopy data.

The downside of metallic nanoparticles is their instability due to oxidation and coagulation processes. These reasons make the use of stabilizers that ensure controlled passivation of nanoparticles relevant. To study the stability of metal–polymer nanocomposite dispersions, absorption bands were used at 415 nm and 370 nm (Figure S4 in Supplementary Information) for samples with an excess and a deficiency of PAA (Figure 6).

Figure S4 in Supplementary Materials show the intensities of the plasmonic signals of silver nanoparticles depending on the holding time of PEI/PAA-Ag dispersions with the ratios 1PEI/3PAA and 3PEI/1PAA. Analysis of UV-Vis spectra does not show noticeable change in the content of metal–polymer hybrids during seven days of storage, which indicates their relatively high stability. The obtained data correlate with the results of electrophoretic mobility measurements, the values of which are −3.6 ± 0.3 (µm·s^−1^)/(V·cm^−1^) and 1.22 ± 0.13 (µm·s^−1^)/(V·cm^−1^) for hybrid colloids with an excess of PAA and PEI, respectively. Thus, as in IPEC, the sign of electrophoretic mobility is determined by the component present in excess. Thus, electrostatic repulsion of excess links of macromolecules ensures the stability of colloids. Further storage leads to a gradual decrease in signal intensity. The observed precipitation indicates that there is a gradual loss of the aggregative stability of the nanomaterials.

**Figure 7:**
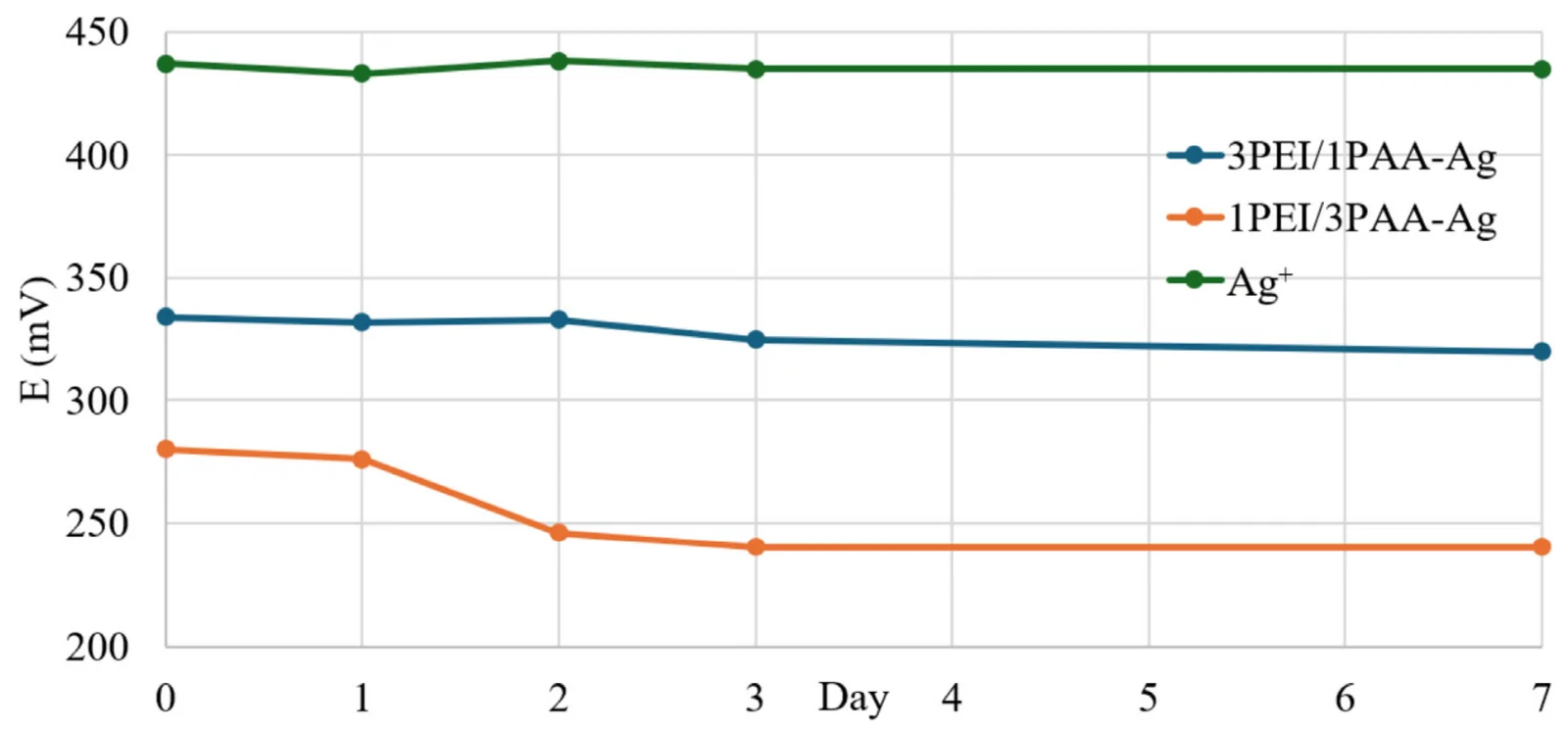
Monitoring of Ag^+^ content for 3PEI/1PAA-Ag, 1PEI/3PAA-Ag dispersions according potentiometry data.

Another aspect of using metal–polymer composites is the activity and potential toxicity of silver nanoparticles due to the release of silver ions. Figure 7 shows monitoring data for the 3PEI/1PAA-Ag and 1PEI/3PAA-Ag systems, obtained using potentiometry.

The total concentration of atoms and ions in terms of the total volume of the solution was 1.6 × 10^−3^ M. The conversion between the activity of silver ions and the potential of the silver electrode was carried out using the formula E = E_0_ + b log_10_ a(Ag^+^) based on the calibrated data. For the 3PEI/1PAA-Ag and 1PEI/3PAA-Ag complexes, the content of ions not bound by ligands of the macromolecules were 3 × 10^−5^ M and 5 × 10^−6^ M, respectively. Thus, the concentrations of unbound ions in colloidal solutions are hundreds of times lower than in a silver nitrate solution. The result obtained is explained by the fact that IPEC PAA-PEI are able to absorb metal ions from solutions with their content of 10^−5^ M and below [38]. Storage of nanohybrid solutions for seven days did not lead to the release of silver ions from the nanoparticles, which confirms their stability. On the contrary, there was a decrease in the concentration of Ag^+^ aqua complexes. Functionally, IPEC groups can act as reducing agents for silver ions, which may explain the decrease in their concentration in solution. It is valid in solutions of metal–polymer complexes PEI-PAA-Ag^+^ that the gradual formation of nanoparticles is observed (Figure S5 in Supplementary Information).

### 3.2. Fungicidal Properties of PEI/PAA Complexes and Ternary PEI/PAA-Ag Nanoparticle Hybrids

#### 3.2.1. Fungicidal Activity in Suspensions of Macromolecular Complexes and Molds

First and foremost, the tests were carried out in suspensions of macromolecular complexes and mold. The present study examined the antifungal properties of PEI/PAA-based macromolecular complexes. The test cultures included six mold strains isolated from food-processing facilities and identified as *Penicillium gladioli*, *P. rugulosum*, *P. polonicum*, *Cladosporium pseudochalastosporoides*, *Botrytis cinerea* and *Aspergillus wentii*. Among the isolated strains, *P. gladioli* is a phytopathogenic species associated with bulbous plants [62,63]. Its isolation from a food-processing facility may indicate contamination of plant-derived raw materials because its presence in production environments may be associated with the processing of products contaminated with soil or plant residues [64]. *Penicillium rugulosum* is of interest as a producer of hepatotoxic metabolites [65]. *P. polonicum* is particularly important because this species, formerly included in the *P. aurantiogriseum* complex, produces several secondary metabolites, including verruculogen, penicilloic acid and nephrotoxic compounds [66]. Species of the genus *Cladosporium* frequently act as secondary contaminants of food products during storage, particularly under conditions of elevated humidity [67]. *Botrytis cinerea* is a phytopathogen with a very broad host range and causes substantial economic losses in the field and during storage [68]. Its pathogenicity is associated with a combination of virulence factors, including cell-wall-degrading enzymes such as pectinases, cellulases and xylanases, phytotoxic metabolites, oxalic acid and the induction of oxidative stress in host tissues [69]. *Aspergillus wentii* has a dual role in the food industry. On the one hand, it is used biotechnologically as a producer of enzymes and citric acid. On the other hand, this cosmopolitan soil fungus is widely distributed on organic substrates and is a common contaminant of grain, nuts and other plant-derived products, particularly under conditions of high humidity [70]. Thus, the taxonomic analysis of the isolates showed that they represented three ecological and functional groups: potentially toxigenic species (*P. rugulosum* and *P. polonicum*), phytopathogens (*P. gladioli* and *B. cinerea*) and saprotrophic contaminants (*C. pseudochalastosporoides* and *A. wentii*). The results of the systematic evaluation of fungicidal activity PEI, PEI/PAA IPECs and their metal–polymer nanohybrids are presented in Table 1.

The results demonstrated a pronounced dependence of antifungal activity on the composition of the polymer formulations. The initial PEI solution inhibited most of the tested strains but did not show detectable antifungal activity against *A. wentii*. Cationic PEI macromolecules may interact with anionic phospholipids and other negatively charged components of fungal cell membranes [36], thereby destabilizing the cell envelope. However, PEI was ineffective against *A. wentii*. This resistance may be associated with the specific structural and physicochemical properties of the Aspergillus cell envelope, including hydrophobic surface components that may reduce the adsorption of PEI [71]. The resistance of Aspergillus indicates the need to enhance the treatment efficacy in order to overcome its protective mechanisms.

The suspensions of a complex with an excess of PEI (3PEI/1PAA) exhibited a substantially narrower activity spectrum than the PEI solution. The formulation retained high activity only against *P. rugulosum* and *B. cinerea.* The complex 1PEI/3PAA did not show detectable antifungal activity against the tested strains. These results contrast with the high activity of free PEI and confirm the critical role of positively charged units.

The differences in strain susceptibility of the fungi to PEI and its complexes were notable even within related taxonomic groups. For example, *P. rugulosum* was more susceptible to the complex, whereas *P. gladioli* and *P. polonicum* remained viable under the same conditions. This finding indicates that the antifungal activity was determined not only by the general properties of the polymer system but also by structural and functional characteristics of the cell envelope of each strain.

The decrease in activity of the IPECs may be caused by both a decrease in the number of accessible cationic PEI groups and changes in the morphology of the colloidal particles. In PEI solutions, the macromolecules may interact efficiently with the microorganism surface. During IPEC formation, the interaction between PEI and PAA blocks a substantial fraction of the positively charged amino groups responsible for binding to microbial surfaces. Consequently, the electrostatic interaction between the IPEC particles and fungal cell envelopes is weakened, reducing the biocidal effect. However, particle morphology may be an even more important factor. The 3PEI/1PAA complex formed particles approximately 210 nm in diameter with a hydrophobic core approximately 210 nm in diameter, whereas excess PEI units were localized in the outer region of the particles (Figure 1), as indicated by the electrophoretic mobility data (more detailed information in Supplementary Materials, Table S1). This organization may preserve only limited antifungal activity because the hydrophobic core and steric restrictions of cationic groups hinder contact between the active sites and the fungal surface. In other words, even in the presence of excess PEI, its mobility and accessibility for interaction with the cell envelope are limited. In the 1PEI/3PAA complex, virtually all positively charged PEI units are associated with PAA within the hydrophobic region of the complex, whereas negatively charged PAA groups predominate in the outer region of the particle. The morphology of the complex with an excess of PAA is the reason for the inactivity of this complex. Overall, the suspension assays showed (Table 1) that the PEI solution and both the 3PEI/1PAA and 1PEI/3PAA IPEC dispersions did not provide complete inhibition of all tested molds.

Modification of the complexes with silver nanoparticles resulted in a pronounced enhancement of their fungicidal activity. However, the extent of this enhancement depended on the composition of the parent complex. The nanohybrid with an excess of PEI inhibited *P. gladioli*, *P. rugulosum*, *P. polonicum*, *C. pseudochalastosporoides* and *B. cinerea* but remained ineffective against *A. wentii*. Thus, the incorporation of silver nanoparticles into this complex substantially broadened its spectrum of fungicidal activity compared with the unmodified 3PEI/1PAA IPEC; however, it did not enable inactivation of the most resistant strain. The most pronounced effect was observed for the nanohybrid with an excess of PAA, which ensured complete killing of all six tested mold strains, including *A. wentii*. This result is particularly noteworthy because the parent complex with the same component ratio, 1PEI/3PAA, showed no detectable fungicidal activity. Therefore, in this system, the incorporation of silver nanoparticles was the decisive factor responsible for the development of the material’s biocidal properties.

At first glance, it may appear paradoxical that the highest antifungal activity was observed for the nanocomposite with an excess of the anionic PAA component. However, this result is consistent with previous data showing higher antibacterial activity for nanocomposites of this composition than for PEI-rich PEI/PAA–Ag systems. A possible explanation is the mutual passivation of polycations and silver nanoparticles in systems with a PEI-rich systems [26]. Strong interactions between the nitrogen-containing groups of PEI and the silver surface may block the essential fraction of the PEI units and simultaneously reduce the accessibility of the silver nanoparticles, thereby weakening their antimicrobial action. In irradiated PEI solutions, smaller nanoparticles are formed compared to solutions of PAA [26], which verifies the strong interaction between PEI macromolecules and nanoparticles. In the system with a lower proportion of PEI, this passivation may be less pronounced, allowing the silver nanoparticles to retain greater intrinsic antifungal activity. PAA and PEI units are strong ligands for metal ions; in the dispersions of nanohybrids, the concentration of Ag^+^ aqua complex is 3 × 10^−5^ M and 5 × 10^−6^ M for 3PEI/1PAA-Ag and 1PEI/3PAA-Ag, respectively. Furthermore, the nanohybrid with an excess of PAA with a minimal content of silver ions exhibits the highest fungicidal activity. This result allows us suppose that in condition of strong complexation macromolecules with silver ions the main contribution to the fungicidal activity is made by the interactions of the nanoparticles with molds, rather than by the release of silver ions. Thus, the results indicate that unmodified PEI/PAA IPECs have low or strain-dependent antifungal activity, whereas modification with silver nanoparticles substantially enhances their efficacy. The most promising formulation was the nanocomposite based on the 1PEI/3PAA-Ag, for which fungicidal activity was detected against all six tested mold strains.

#### 3.2.2. Fungicidal Activity of Macromolecular Complexes Against Preformed and Developing Mold Biofilms

The main advantage of IPEC based on PEI/PAA and their metal–polymer hybrids for the development of antifungal materials is the potential for their use in biocidal coatings [26,27,40,41,42,43,44]. PEI/PAA-based formulations can be applied to both hydrophobic and hydrophilic surfaces, including porous materials, or incorporated into soil [38]. For the initial study of the possibilities of obtaining antifungal surfaces in the present study, antifungal performance was assessed using a glass-fiber-filter screening model treated with polymer dispersions. Therefore, the results should be interpreted as evidence of antifungal potential in a model surface-associated system rather than as direct proof of coating performance on practical substrates.

Accordingly, the antifungal efficacy of selected macromolecular complexes was evaluated using two complementary models: preformed mold biofilms and biofilm formation on polymer-treated surfaces. The study included the 3PEI/1PAA IPEC, metal–polymer hybrids 3PEI/1PAA-Ag and 1PEI/3PAA-Ag containing silver nanoparticles because formulations had previously shown antifungal activity in mold suspensions.

**Figure 8:**
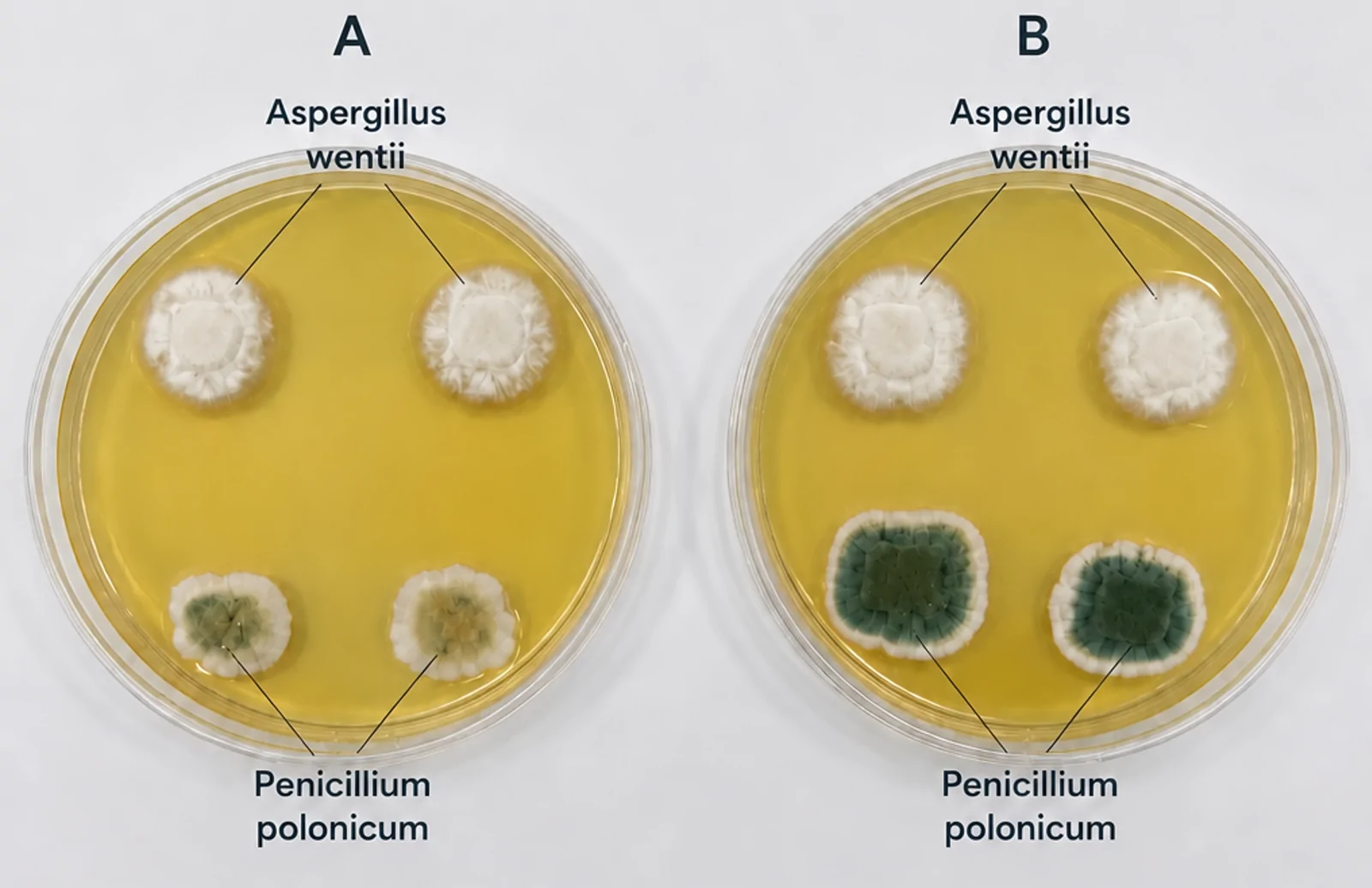
Fungicidal activity of the 1PEI/3PAA-Ag composite against preformed mold biofilms: (**A**) treated sample; (**B**), untreated control.

For systematic evaluation, *P. polonicum and A. wentii* were selected as mold strains characterized by comparatively high resistance to disinfectants [71]. The use of strains belonging to different genera also allowed the activity of the complexes to be compared against molds with potentially different cell-wall structures and antimicrobial susceptibility profiles. Most representative photographs demonstrating the antifungal activity of the polymer formulations against preformed biofilms are shown in Figure 8 (more detailed information in Supplementary Materials, Figure S6).

The results of the evaluation of the metal–polymer composites against preformed biofilms are presented in Table 2. The tested formulations generally showed limited activity against preformed biofilms. In the control samples, the number of viable cells of *P. polonicum* and *A. wentii* was approximately 1.0 × 10^7^ CFU cm^−3^, confirming the formation of a mature biofilm-like structure. The IPEC with composition 3PEI/1PAA showed only moderate activity. Against *P. polonicum*, the viable-cell count decreased to 6.9 × 10^6^ CFU cm^−3^, corresponding to an efficacy of 31%. Against *A. wentii*, no effect was detected, and the viable-cell count remained at the control level. The most pronounced effect was observed for the hybrid of 1PEI/3PAA with silver nanoparticles against *P. polonicum.* The viable-cell count decreased to 1.2 × 10^6^ CFU cm^−3^, corresponding to an efficacy of 88%. The formulation was not effective against *A. wentii* under the conditions used. The hybrid of composition 3PEI/1PAA with silver nanoparticles had only a small effect on the preformed biofilms of either strain. The viable-cell count remained approximately 1.1 × 10^7^ CFU cm^−3^ for *P. polonicum* and 1.0 × 10^7^ CFU cm^−3^ for *A. wentii.* Consequently, no pronounced antifungal effect was detected. These results indicate that preformed mold biofilms are highly resistant to the tested metal–polymer systems. At the concentrations investigated, incorporation of silver nanoparticles did not result in complete eradication of the mature biofilms. The reduction in viable-cell count produced by 3PEI/1PAA against *P. polonicum* was statistically significant (*p* < 0.05), whereas the effect of 1PEI/3PAA-Ag was even more pronounced (*p* < 0.001). In contrast, 3PEI/1PAA-Ag did not differ essentially from the control for either strain. No inhibitory effect was detected against *A. wentii* under the tested conditions.

**Figure 9:**
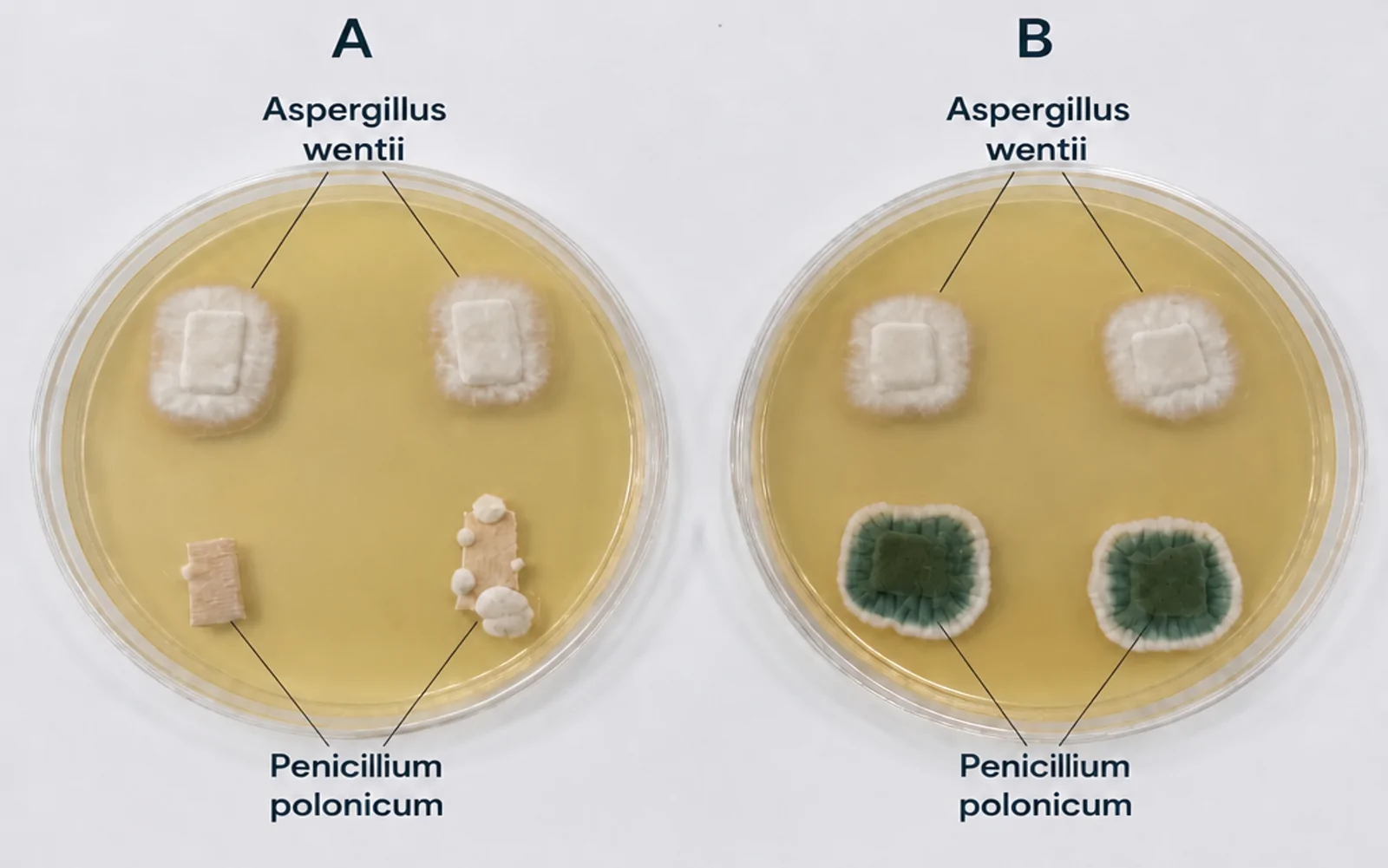
Fungicidal activity of the 1PEI/3PAA-Ag composite against mold biofilm formation: (**A**) treated sample; (**B**), untreated control.

A different pattern was observed (Figure 9; Table 3) when the polymer formulations were applied before biofilm formation (more detailed information in Supplementary Materials, Figure S7).

In the control samples, biofilm formation was extensive for both *P. polonicum* and *A. wentii*, with viable-cell counts reaching approximately 2.1 × 10^7^ CFU cm^−3^. The silver-free IPEC with composition 3PEI/1PAA exhibited strong strain selectivity (Table 1). Against *P. polonicum*, its activity was minimal: the viable-cell count remained approximately 2.0 × 10^7^ CFU cm^−3^, corresponding to an efficacy of only 5%. Against *A. wentii*, however, the formulation showed a strong inhibitory effect, reducing the viable-cell count to 1.0 × 10^5^ CFU cm^−3^ and corresponding to an efficacy of 90%. The highest overall activity was again observed for hybrids of 1PEI/3PAA-Ag. Against *P. polonicum*, the viable-cell count decreased to 2.0 × 10^3^ CFU cm^−3^, corresponding to an efficacy of 99% and near-complete inhibition of biofilm formation. Against *A. wentii*, the activity of this formulation was lower but remained substantial. The viable-cell count decreased to 3.0 × 10^6^ CFU cm^−3^, corresponding to an efficacy of 85%. The hybrids of 3PEI/1PAA-Ag also inhibited biofilm formation, although less effectively. The activity was 42% against *P. polonicum* and 87% against *A. wentii* (Table 3). During biofilm formation, the effect of 3PEI/1PAA against *P. polonicum* was not statistically significant, whereas the reductions observed for 1PEI/3PAA-Ag and 3PEI/1PAA-Ag were considerable. Compared to the control sample, the number of viable *A. wentii* cells decreased greatly in all tested formulations.

All tested formulations significantly reduced the number of viable *A. wentii* cells compared to the control group.

Comparison of the results showed that the metal–polymer composites were substantially more effective at preventing the early stages of adhesion and biofilm formation than at acting on mature biofilm structures. This difference is consistent with the organization of mature biofilms, in which cells are embedded in an extracellular matrix that limits the diffusion of active components and reduces their accessibility to cellular targets. During the early stages of biofilm development, cells are more vulnerable, and surface pretreatment may interfere with spore adhesion, initial attachment and subsequent colony development [72].

## 4. Conclusions

The obtained results indicate the important role of the composition and structural organization of macromolecular complexes and nanohybrids in their biocidal behavior. The fungicidal activity of PAA–PEI complexes is governed by the presence of free positively charged units, with the spatial organization of the complex particles playing a key role. IPECs with an excess of cationogenic PEI exhibit fungicidal activity against certain mold strains. Steric restrictions on the interaction of the external tails of the cationic component with the cell membrane account for the partial loss of fungicidal activity of 3PEI/1PAA colloidal particles compared with single PEI macromolecules. In PAA-rich complexes, the negatively charged units are located in the outer part of the particles, which blocks activity against the studied mold fungi.

Incorporating silver nanoparticles into the IPECs as a filler leads to a dramatic increase in fungicidal activity, consistent with earlier antibacterial results for PEI/PAA-based metal–polymer nanohybrids. The PAA-rich macromolecular complex showed the best antifungal performance against all tested molds. In PEI-rich nanohybrid, considerably smaller nanoparticles were generated; nevertheless, this nanohybrid exhibited lower activity. A plausible explanation for this paradoxical result is the mutual passivation of PEI and the silver nanoparticles caused by strong interaction between the nitrogen-containing units and the nanoparticle surface [26,27].

Of particular interest is the variation in fungal sensitivity among the studied strains, even within the same genus, upon exposure to PEI-based complexes and nanohybrids. This indicates that the response is determined not only by the general properties of the polymer system but also by the structural and functional features of the cell wall of a particular strain. In contrast to their fungicidal activity, the PEI/PAA complexes with either cationogenic or anionogenic unit excess exhibited relatively high antibacterial activity [26], which is attributed to the less stable bacterial membrane compared with that of fungi.

This work was motivated primarily by the goal of creating fungicidal coatings for warehouses and food-production facilities, including hard-to-reach areas, since PEI–PAA complexes allow interactions with both hydrophilic and hydrophobic surfaces to be tuned. Water as a solvent ensures the fire safety and environmental friendliness of the formulation. Importantly, the biofilm experiments demonstrated that these materials were substantially more effective at preventing mold adhesion and biofilm formation than at inactivating mature, preformed biofilms. Thus, under the tested conditions, the PEI/PAA–Ag systems should be regarded primarily as preventive rather than curative antifungal formulations.

This distinction is particularly relevant for prospective surface-treatment and coating applications and should be taken into account in further application-oriented studies. Since the present experiments were performed in a glass-fiber-filter screening model, additional studies on practical substrates—including coating stability, wash resistance, and long-term activity—will be necessary. It is also worthwhile to continue work on introducing the macromolecular complexes into soil, where they would destroy the fungus at the first stage and prevent its re-growth at the second, owing to the binding of the IPECs to soil particles.

## Figures and Tables

**Table 1:** Fungicidal activity of PEI-based macromolecular complexes after 24 h of exposure.

Polymer Complex	*Penicillium gladioli* № 7034	*Penicillium rugulosum* № 7040	*Penicillium polonicum* № 7046	*Cladosporium pseudochalasfosporoides* № 7036	*Botrytis cinerea* № 7045	*Aspergillius wentii* № 7049
PEI	✓	✓	✓	✓	✓	✗
3PEI/1PAA	✗	✓	✗	✗	✓	✗
1PEI/3PAA	✗	✗	✗	✗	✗	✗
3PEI/1PAA-Ag	✓	✓	✓	✓	✓	✗
1PEI/3PAA-Ag	✓	✓	✓	✓	✓	✓

*Note: «* ✓ *»—the presence of fungicidal activity (100% cell death), «* ✗ *»—lack of fungicidal activity (not 100% cell death).*

**Table 2:** Fungicidal activity of the metal–polymer composites against preformed mold biofilms.

№	Polymer Complex	*P* *enicillium polonicum*		*A* *spergillus wentii*	
		Number of Mold Fungi, CFU cm^−3^	Activity, %	Number of Mold Fungi, CFU cm^−3^	Activity, %
1	Control sample	(1.0 ± 0.20) × 10^7^	-	(1.0 ± 0.10) × 10^7^	-
2	3PEI/1PAA	(6.9 ± 0.14) × 10^6^ *	31	(1.5 ± 0.20) × 10^7^ ns	-
3	1PEI/3PAA-Ag	(1.2 ± 0.20) × 10^6^ ***	88	(1.0 ± 0.20) × 10^7^ ns	-
4	3PEI/1PAA-Ag	(1.1 ± 0.36) × 10^7^ ns	-	(1.0 ± 0.15) × 10^7^ ns	-

Data are presented as mean ± SD (*n* = 3). Statistical significance of differences from the untreated control: ns, not significant; * *p* < 0.05; *** *p* < 0.001.

**Table 3:** Fungicidal activity of the metal–polymer composites during mold biofilm formation.

№	Polymer Complex	*Penicillium polonicum*		*Aspergillus wentii*	
		Number of Mold Fungi, CFU cm^−3^	Activity, %	Number of Mold Fungi, CFU cm^−3^	Activity, %
1	Control sample	(2.1 ± 0.21) × 10^7^	-	(2.1 ± 0.20) × 10^7^	-
3	3PEI/1PAA	(2.0 ± 0.10) × 10^7^ ns	5	(1.0 ± 0.10) × 10^5^ ***	90
2	1PEI/3PAA-Ag	(2.0 ± 0.30) × 10^3^ ***	99	(3.0 ± 0.26) × 10^6^ ***	85
4	3PEI/1PAA-Ag	(1.2 ± 0.17) × 10^7^ **	42	(2.6 ± 0.20) × 10^6^ ***	87

Data are presented as mean ± SD (*n* = 3). Statistical significance of differences from the untreated control: ns, not significant; ** *p* < 0.01; *** *p* < 0.001.

## Data Availability

The original contributions presented in this study are included in the article/Supplementary Material. Further inquiries can be directed to the corresponding author.

## Supplementary Materials

The following supporting information can be downloaded at https://www.mdpi.com/article/10.3390/polym18212772/s1, Scheme S1: Investigation the fungicide properties of polymer complexes; Figure S1: FTIR spectra of complexes (the pH value of initial solution is 6.5); Figure S2: The TEM image of UV irradiated dispersion of 1PEI/3PAA-Ag^+^. Irradiation time 20 min; Figure S3: The TEM image of UV irradiated dispersion of 3PEI/1PAA-Ag^+^. Irradiation time 20 min; Figure S4: Colloidal stability in dispersion 1PEI/3PAA-Ag and 3PEI-1PAA-Ag according UV-Vis spectra; Figure S5: Colloidal stability in dispersion 1PEI/3PAA-Ag^+^ and 3PEI-1PAA-Ag^+^ according UV-Vis spectra; Figure S6: Fungicidal activity of the metal–polymer composites against preformed mold biofilms: (I) 3PEI/1PAA; (II) 3PEI/1PAA-Ag, UV-irradiated. “A”, treated sample; “B”, untreated control; Figure S7: Fungicidal activity of the metal–polymer composites during mold biofilm formation: (I) 3PEI/1PAA; (II) 3PEI/1PAA-Ag, UV-irradiated. “A”, treated sample; “B”, untreated control; Table S1: DLS data IPEC complexes of PEI/PAA; Table S2: Electrophoretic mobility for colloidal particles of 1PEI/3PAA-Ag and 3PEI-1PAA-Ag.

## Abbreviations

The following abbreviations are used in this manuscript:

IPECs	Interpolyelectrolyte complexes
PEI	polyethyleneimine
PAA	polyacrylic acid
