# Supplementary material for: Fungicidal Behavior of Polyethyleneimine—Polyacrylic Acid Complexes and Hybrids with Silver Nanoparticles: The Role of Filler and Morphology
Source: Polymers (Basel). 2026 Nov 6;18(21):2772. doi: 10.3390/polym18212772 (PMC13765875; doi:10.3390/polym18212772)
Supplement: Supplementary file 1 [file polymers-18-02772-s001.zip › polymers-4580570-supplementary.pdf]

Fungicidal behaviour of polyethylenimine – polyacrylic acid complexes and hybrides with silver nanoparticles: the role of filler and morphology (Supplementary Information)

Klimov Dmitry I.[1,2]; Vinogradov Rodion.A.[1,2]; Semenova Anastasia A.[3]; Yushina Yulia K.[3]; Mahova Anzhelika A.[3]; Zaiko Elena V.[3]; Grudistova Maria A.[3]; Zezina Elena A.[1]; Zharikov Alexey A.[1]; Zezin Alexey A.[1,3]

[1]Department of Chemistry, M.V. Lomonosov Moscow State University, Leninskie gory 1-3, Moscow 119991, Russia
[2]Enikolopov Institute of Synthetic Polymeric Materials, Russian Academy of Sciences, Profsoyuznaya st. 70, Moscow 117393, Russia
[3]V.M. Gorbatov Federal Research Center for Food Systems, Talalikhina st., 26, Moscow 109316, Russia

Table of contents

S1. Methodology for studying the fungicidal effect of polymer complexes ... 2

Scheme S1. Investigation the fungicide properties of polymer complexes ... 2

S2. Complete steps for synthesis of PEI/PAA IPECs, silver containing complexes and silver nanoparticles containing hybrids ... 3

S3. Characterization of PEI/PAA IPEC and PEI/PAA-Ag$^+$ complexes ... 5

Figure S1. FTIR spectra of complexes (the pH value of initial solution is 6.5) ... 5

Table S1. DLS data IPEC complexes of PEI/PAA ... 6

S4. Characterization of Ag nanoparticles ... 7

Figure S2. The TEM image of UV irradiated dispersion of 1PEI/3PAA-Ag$^+$. Irradiation time 20 min ... 7

Figure S3. The TEM image of UV irradiated dispersion of 3PEI/1PAA-Ag$^+$. Irradiation time 20 min ... 8

Figure S4. Colloidal stability in dispersion 1PEI/3PAA-Ag and 3PEI-1PAA-Ag according UV-Vis spectra. ... 9

Figure S5. Colloidal stability in dispersion 1PEI/3PAA-Ag$^+$ and 3PEI-1PAA-Ag$^+$ according UV-Vis spectra. ... 9

Table S2. Electrophoretic mobility for colloidal particles of 1PEI/3PAA-Ag and 3PEI-1PAA-Ag. . 9

S5. Fungicidal activity of PEI/PAA complexes and PEI/PAA-Ag ... 10

Figure S6. Fungicidal activity of the metal–polymer composites against preformed mold biofilms: (I) 3PEI/1PAA; (II) 3PEI/1PAA-Ag, UV-irradiated. “A”, treated sample; “B”, untreated control.. 10

Figure S7. Fungicidal activity of the metal–polymer composites during mold biofilm formation: (I) 3PEI/1PAA; (II) 3PEI/1PAA-Ag, UV-irradiated. “A”, treated sample; “B”, untreated control. ... 10

S1. Methodology for studying the fungicidal effect of polymer complexes

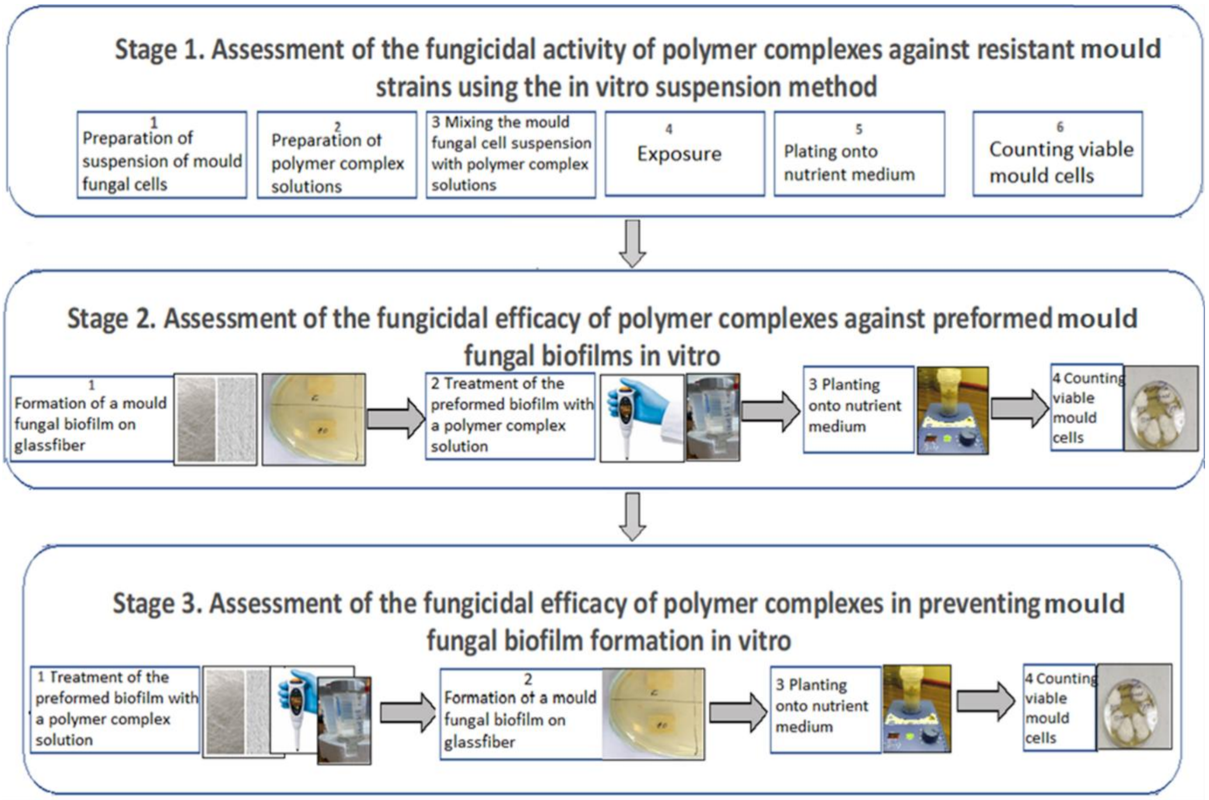

Scheme S1. Investigation the fungicide properties of polymer complexes

S2. Complete steps for synthesis of PEI/PAA IPECs, silver containing complexes and silver nanoparticles containing hybrids

Preparation of 100 mL of a 0.1% IPEC suspension 1PEI/3PAA:

1. Prepare 40 mL of a 0.2% aqueous solution of the «host polymer» (present in excess in the final composition) by dissolving 0.23 mL of a 35% aqueous PAA solution in 39.77 mL of water. Adjust the pH of the system to 6 using KOH.

2. Prepare 40 mL of a 0.055% aqueous solution of the «guest polymer» (present in a deficient amount in the final composition) by dissolving 0.07 mL of a 30% aqueous PEI solution in 39.93 mL of distilled water. Adjust the pH of the system to 6 using H_2SO_4.

3. To prevent the formation of a macroscopic precipitate, add the aqueous solution of the guest polymer (PEI) to the host polymer solution (PAA) under constant stirring. This yields 80 mL of a 0.125% aqueous suspension of the complex, in which three functional groups of the host polymer (PAA) correspond to one functional group of the guest polymer (PEI), designated as «1PEI/3PAA».

4. To prepare a 0.1% aqueous suspension of the metal-free IPEC, add 20 mL of distilled water to the suspension obtained in Step 3.

5. To prepare the 1PEI/3PAA–Ag hybrid, add 20 mL of a 0.13% aqueous $AgNO_3$ solution to the suspension obtained in Step 3. This yields a 0.1% aqueous suspension of the 1PEI/3PAA–Ag^+ complex. Thus, one Ag^+ ion in the complex corresponds to two functional groups of the guest polymer (PEI).

6. To reduce silver ions and form nanoparticles, irradiate the 0.1% 1PEI/3PAA–Ag^+ suspension obtained in Step 5 with UV radiation once for 20 min, producing the «1PEI/3PAA–Ag» hybrid.

Preparation of 100 mL of a 0.1% IPEC Suspension 3PEI/1PAA:

1. Prepare 40 mL of a 0.16% aqueous solution of the host polymer by dissolving 0.22 mL of a 30% aqueous PEI solution in 39.78 mL of distilled water. Adjust the pH of the system to 6 using H_2SO_4.

2. Prepare 40 mL of a 0.1% aqueous solution of the guest polymer by dissolving 0.125 mL of a 35% aqueous PAA solution in 39.875 mL of water. Adjust the pH of the system to 6 using KOH.

3. To prevent the formation of a macroscopic precipitate, add the aqueous solution of the guest polymer (PAA) to the host polymer solution (PEI) under constant stirring. This yields 80 mL of a 0.125% aqueous suspension of the complex, in which three functional groups of the host polymer (PEI) correspond to one functional group of the guest polymer (PAA), designated as «3PEI/1PAA».

4. To prepare a 0.1% aqueous suspension of the metal-free IPEC, add 20 mL of distilled water to the suspension obtained in Step 3.

5. To prepare the 3PEI/1PAA–Ag hybrid, add 20 mL of a 0.13% aqueous $AgNO_3$ solution to the suspension obtained in Step 3. This yields a 0.1% aqueous suspension of the 3PEI/1PAA–Ag^+ complex. Thus, one Ag^+ ion in the complex corresponds to 2.5 functional groups of the guest polymer (PAA).

6. To reduce silver ions and form nanoparticles, irradiate the 0.1% 3PEI/1PAA–Ag^+ suspension obtained in Step 5 with UV radiation once for 20 min, producing the «3PEI/1PAA–Ag» hybrid.

S3. Characterization of PEI/PAA IPEC and PEI/PAA-Ag^+ complexes

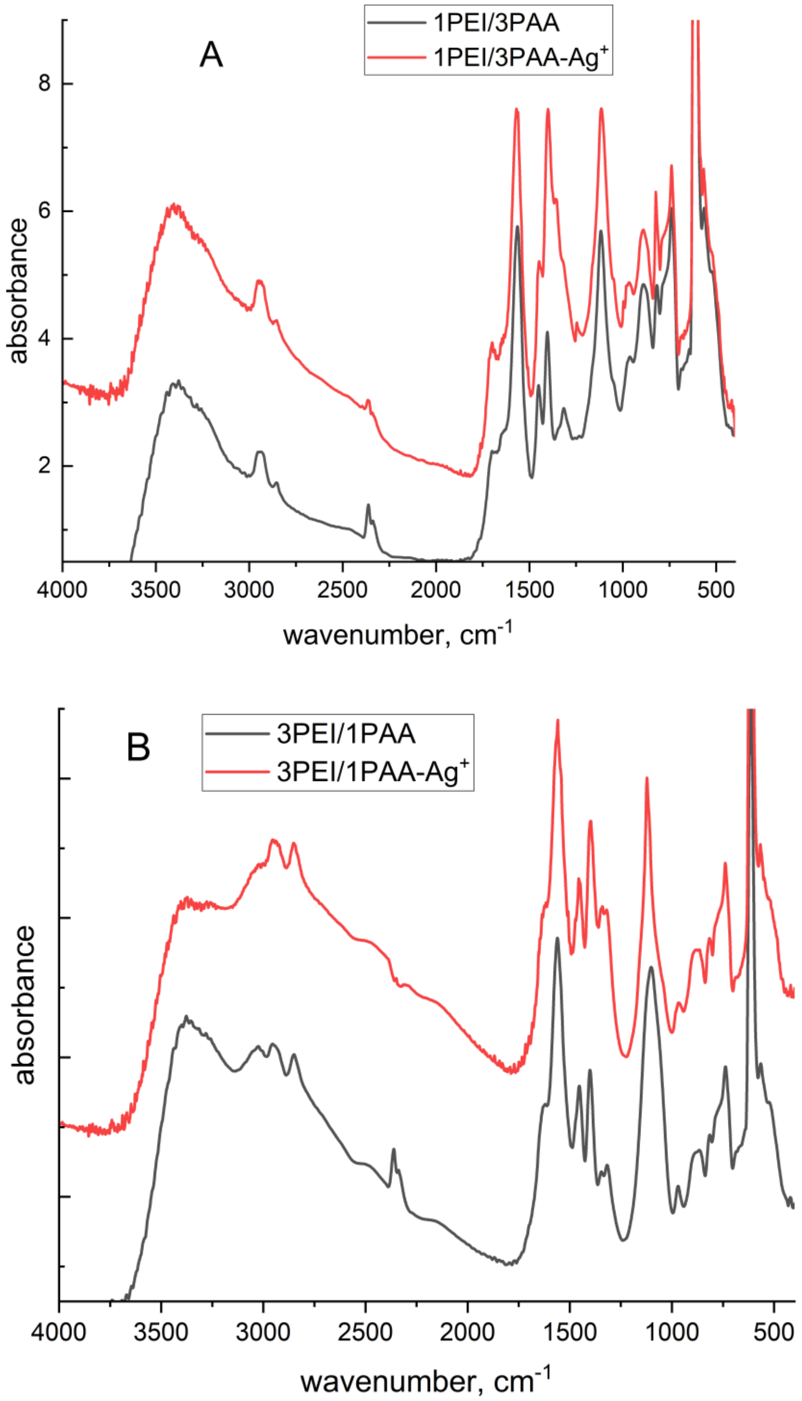

Figure S1. FTIR spectra of IPECs and metal polymer complexes (the pH value of initial solution is 6.5)

Table S1. DLS data IPEC complexes of PEI/PAA

System	D_{ef}, nm	PDI	EPM, (µm s^{-1})/(V cm^{-1})
1PEI/3PAA	130	0.171	-2.97±0.14
3PEI/1PAA	210	0.192	+1.56±0.11

S4. Characterization of Ag nanoparticles

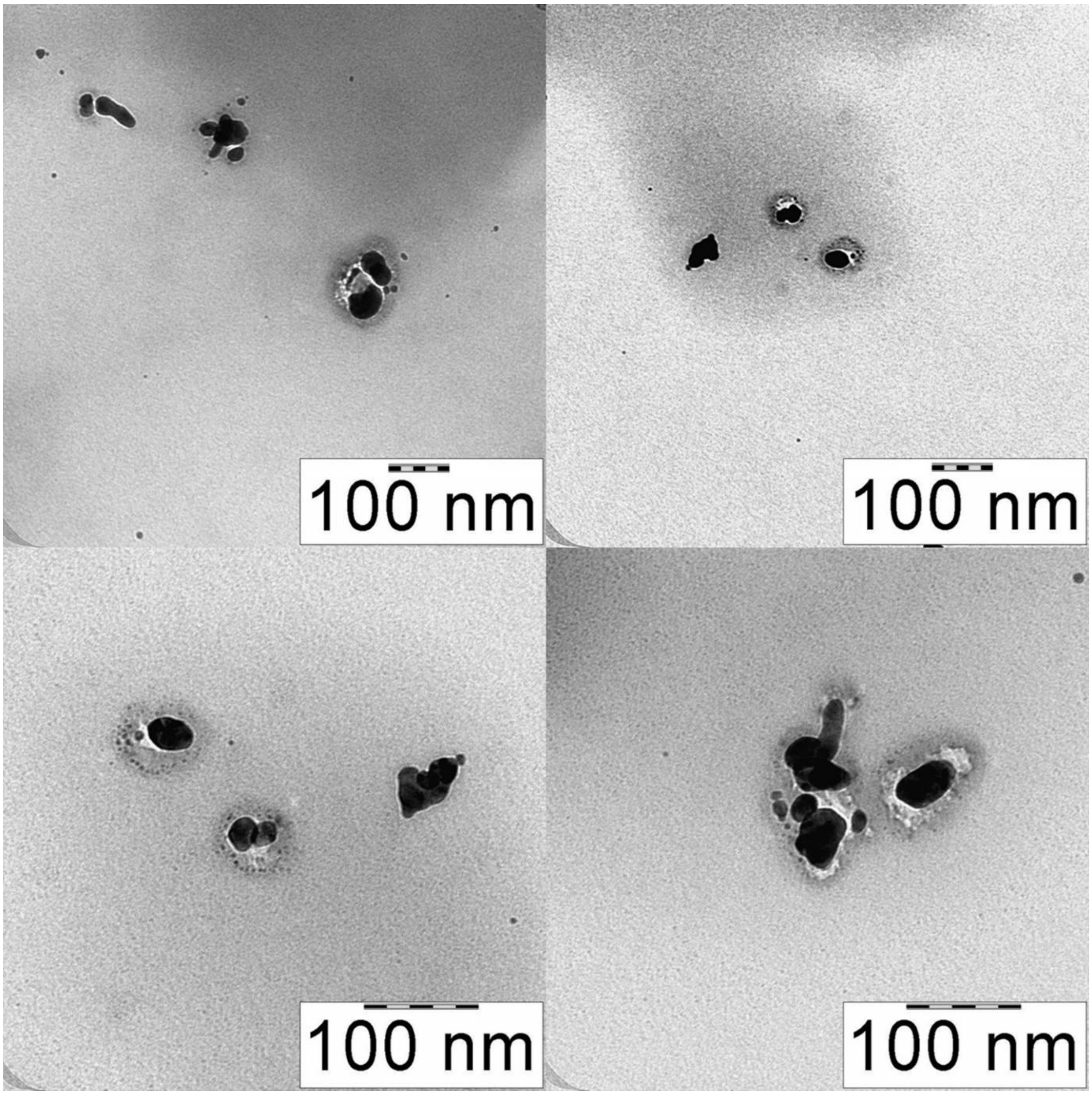

Figure S2. The TEM image of UV irradiated dispersion of 1PEI/3PAA-Ag^{+}. Irradiation time 20 min.

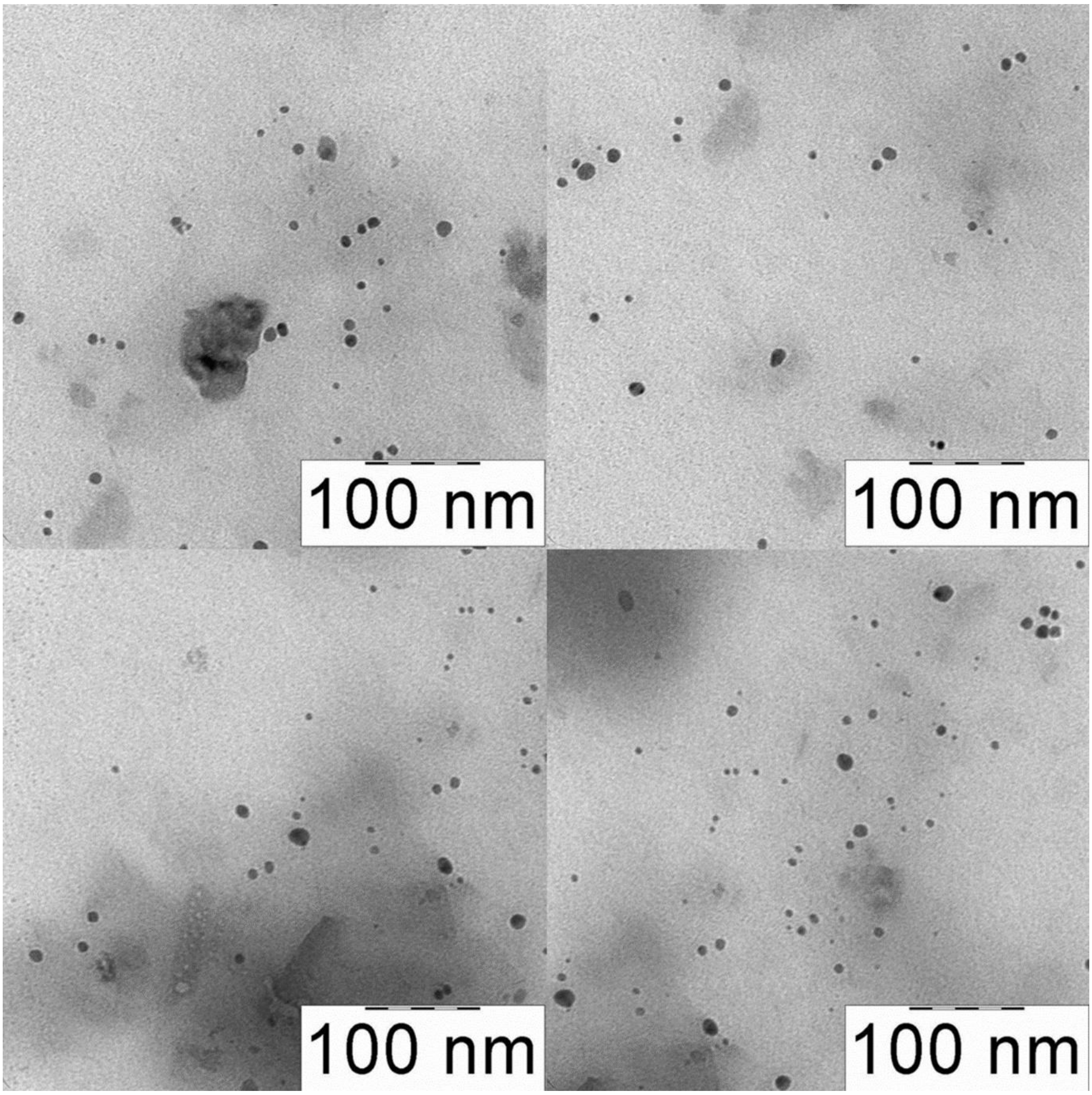

Figure S3. The TEM image of UV irradiated dispersion of 3PEI/1PAA-Ag^{+}. Irradiation time 20 min.

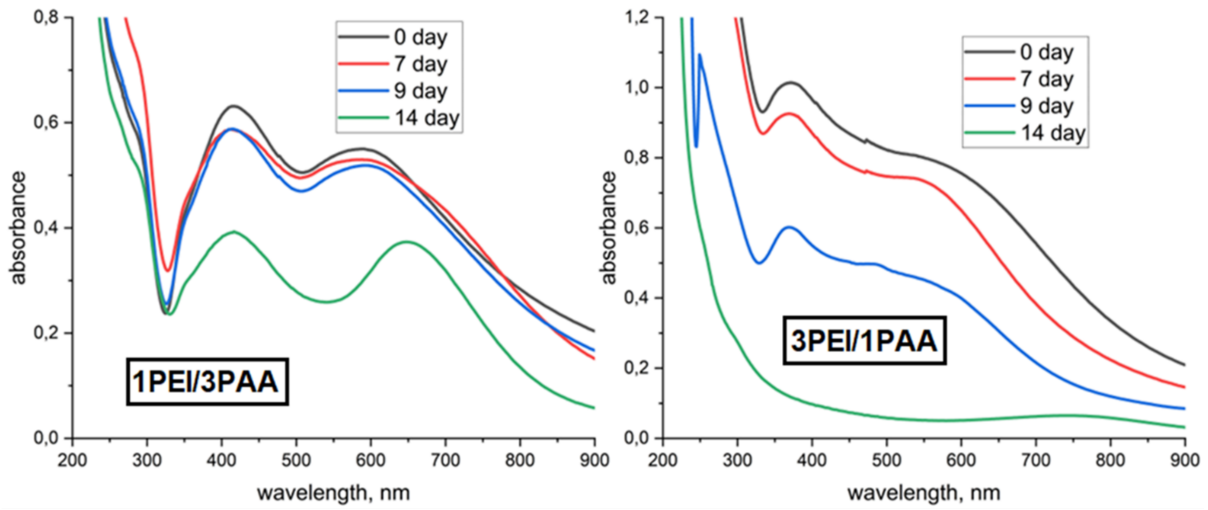

Figure S4. Colloidal stability in dispersion 1PEI/3PAA-Ag and 3PEI-1PAA-Ag according UV-Vis spectroscopy.

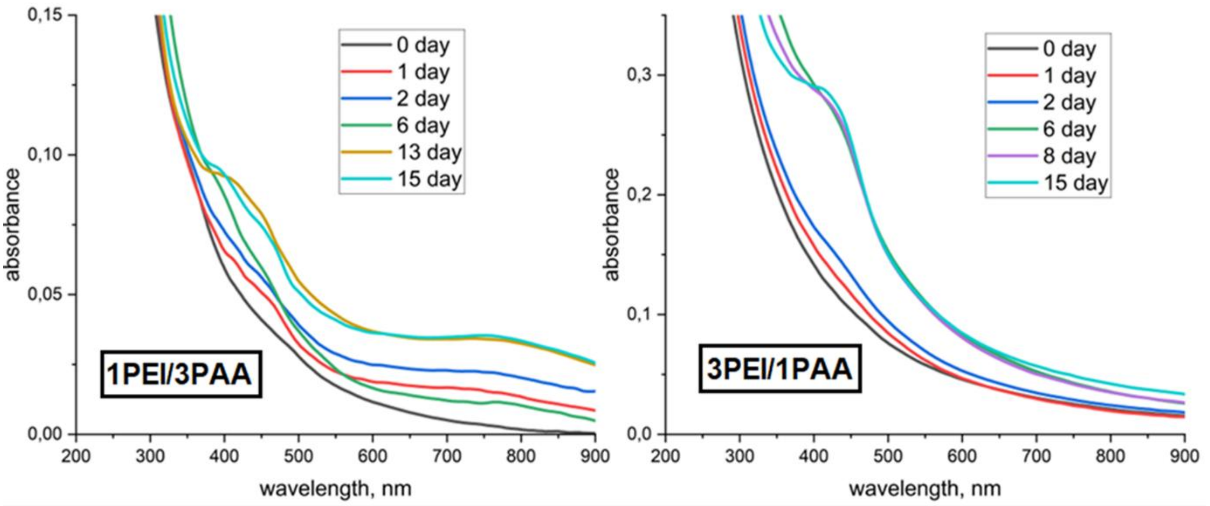

Figure S5. Colloidal stability in dispersion 1PEI/3PAA-Ag^+ and 3PEI-1PAA-Ag^+ according UV-Vis spectroscopy.

Table S2. Electrophoretic mobility for colloidal particles of 1PEI/3PAA-Ag and 3PEI-1PAA-Ag.

System	EPM, (μm s^{-1})/(V cm^{-1})
1PEI/3PAA-Ag	-3.6±0.3
3PEI/1PAA-Ag	+1.22±0.13

S5. Fungicidal activity of PEI/PAA complexes and PEI/PAA-Ag

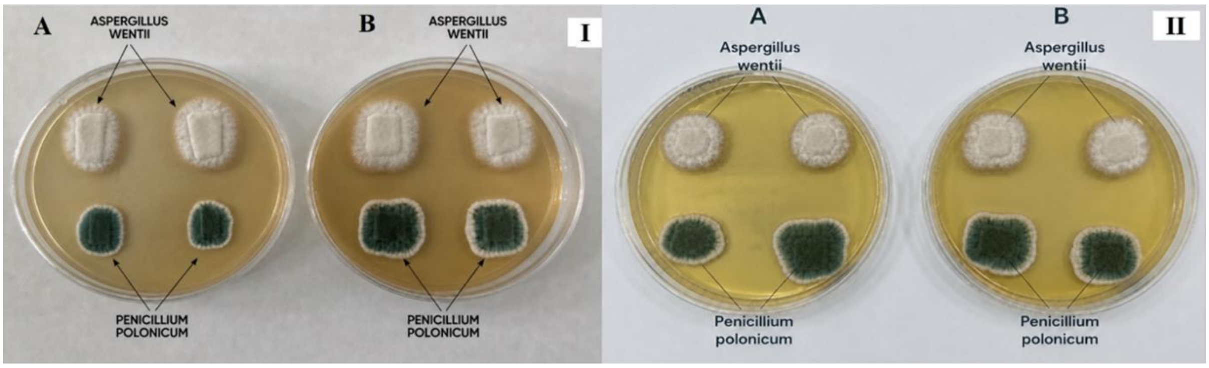

Figure S6. Fungicidal activity of the metal–polymer composites against preformed mold biofilms: (I) 3PEI/1PAA; (II) 3PEI/1PAA-Ag, UV-irradiated. “A”, treated sample; “B”, untreated control.

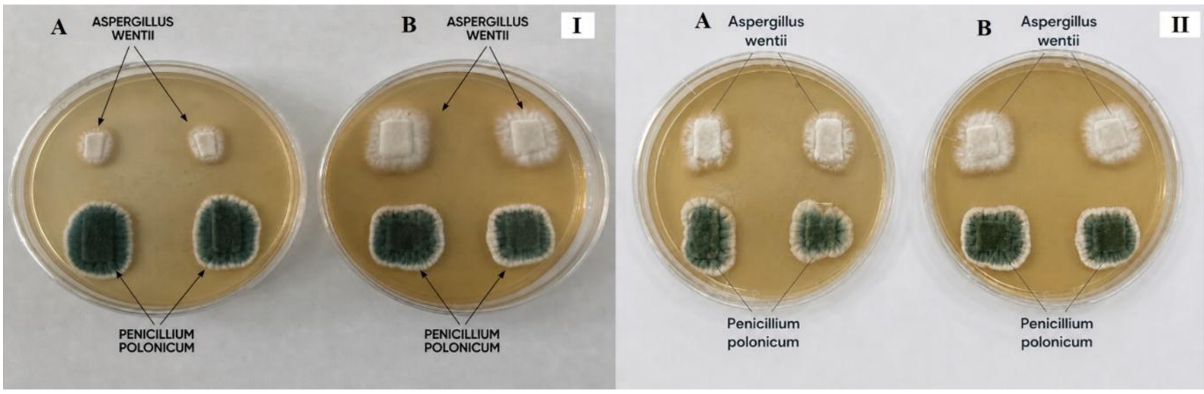

Figure S7. Fungicidal activity of the metal–polymer composites during mold biofilm formation: (I) 3PEI/1PAA; (II) 3PEI/1PAA-Ag, UV-irradiated. “A”, treated sample; “B”, untreated control.
